# A pH-triggered N-oxide polyzwitterionic nano-drug loaded system for the anti-tumor immunity activation research

**DOI:** 10.1186/s12951-024-02677-0

**Published:** 2024-07-16

**Authors:** Yan Zhao, Yuansong Bai, Mei Li, Xin Nie, Hao Meng, Shimizu Shosei, Linlin Liu, Qingbiao Yang, Meili Shen, Yapeng Li

**Affiliations:** 1 Department of Medical Oncology, China-Japan Union Hospital of Jilin University, Changchun, Jilin 130033 China; 2 Stroke center, Jilin Provincial Electric Power Hospital, Changchun, Jilin 130022 China; 3 Department of Radiation Oncology, China-Japan Union Hospital of Jilin University, Changchun, Jilin 130033 China; 4 Pediatric Radiation Therapy Center/Pediatric Proton Beam Therapy Center, University of Tsukuba Hospital, Tsukuba, 3050005 Japan; 5 Hebei Yizhou Cancer Hospital, Zhuozhou, Hebei 072750 China; 6 Key Laboratory of Special Engineering Plastics Ministry of Education, College of Chemistry, Jilin University, Changchun, Jilin 130012 China

**Keywords:** Triple negative breast cancer, Cur, Rg3, Immunogenic cell death, pH response, Nanomicelles

## Abstract

Triple negative breast cancer (TNBC) has the characteristics of low immune cell infiltration, high expression of tumor programmed death ligand 1 (PD-L1), and abundant cancer stem cells. Systemic toxicity of traditional chemotherapy drugs due to poor drug selectivity, and chemotherapy failure due to tumor drug resistance and other problems, so it is particularly important to find new cancer treatment strategies for TNBC with limited treatment options. Both the anti-tumor natural drugs curcumin and ginsenoside Rg3 can exert anti-tumor effects by inducing immunogenic cell death (ICD) of tumor cells, reducing PD-L1 expression, and reducing cancer stem cells. However, they have the disadvantages of poor water solubility, low bioavailability, and weak anti-tumor effect of single agents. We used vinyl ether bonds to link curcumin (Cur) with N-O type zwitterionic polymers and at the same time encapsulated ginsenoside Rg3 to obtain hyperbranched zwitterionic drug-loaded micelles OPDEA-PGED-5HA@Cur@Rg3 (PPH@CR) with pH response. In vitro cell experiments and in vivo animal experiments have proved that PPH@CR could not only promote the maturation of dendritic cells (DCs) and increase the CD4^+^ T cells and CD8^+^ T cells by inducing ICD in tumor cells but also reduce the expression of PD-L1 in tumor tissues, and reduce cancer stem cells and showed better anti-tumor effects and good biological safety compared with free double drugs, which is a promising cancer treatment strategy.

**Supplementary Information:**

The online version contains supplementary material available at 10.1186/s12951-024-02677-0.

## Introduction

Breast cancer is the most common malignant tumor in women, and triple negative breast cancer (TNBC) in breast cancer is the main reason for immune escape, tumor metastasis, and tumor treatment resistance due to its low immune cell invasion, high expression of programmed death ligand 1 (PD-L1) protein and abundance of cancer stem cells [1–3]. Immunogenic cell death (ICD) is an immune-related form of cell death which can activate anti-tumor immune response through the release of damage-associated molecular patterns (DAMPs) [4]. PD-L1 is a major co-inhibitory checkpoint protein that is highly expressed in various cancers and can bind to programmed cell death 1 (PD-1) on T cells to control T cell activity and is currently the main cause of immune escape of tumor cells [5]. While cancer stem cells play an important role in tumorigenesis and tumor biology and are a major cause of tumor metastasis and resistance to therapy [6, 7]. Therefore, inducing ICD in tumor cells, reducing PD-L1 protein expression and cancer stem cells are beneficial to activate and restore anti-tumor immunity, control cancer cell metastasis and overcome drug resistance.

Chemotherapy is still the cornerstone of the treatment of TNBC due to the lack of effective therapeutic targets. And it is particularly important to find anti-tumor treatment alternatives with high treatment selectivity and few side effects due to the systemic toxicity, low specificity and high resistance rate of traditional chemotherapy drugs. Natural products have the advantages of availability, low price, and low toxicity [8], and can exert anti-tumor activity through a variety of mechanisms. Such as curcumin (Cur) and ginsenoside Rg3 extracted from natural products which are ideal anti-tumor natural products that can exert anti-tumor effects by inducing ICD in tumor cells, reducing the expression of tumor PD-L1 protein, and reducing cancer stem cells [9–11]. Unfortunately, the two drugs have the characteristics of poor water solubility, low bioavailability, and weak anti-tumor effect of single agents, so finding strategies to overcome these shortcomings is the key to give full play to the anti-tumor effects of the two drugs [12, 13].

Nano-drug delivery technology can enable efficient drug delivery and enhance anti-tumor efficacy by loading multiple drugs [14]. Zwitterionic polymers have attracted widespread attention due to their high hydrophilicity, biocompatibility, and negligible immunogenicity, which making them ideal drug delivery vehicles [15]. Among them, N-O zwitterionic polymers have been proven to have good biocompatibility, transcytosis and trigger the rapid uptake of drugs by tumor cells and can achieve efficient delivery of anti-tumor drugs [16]. While the click reaction of acylalkyne monomers with phenolic drugs is used to construct hyperbranched zwitterionic polymer nanocarriers, in which the vinyl ether bonds formed can be decomposed at low pH and is a very effective method for pH-responsive drug delivery [17].

**Scheme 1:**
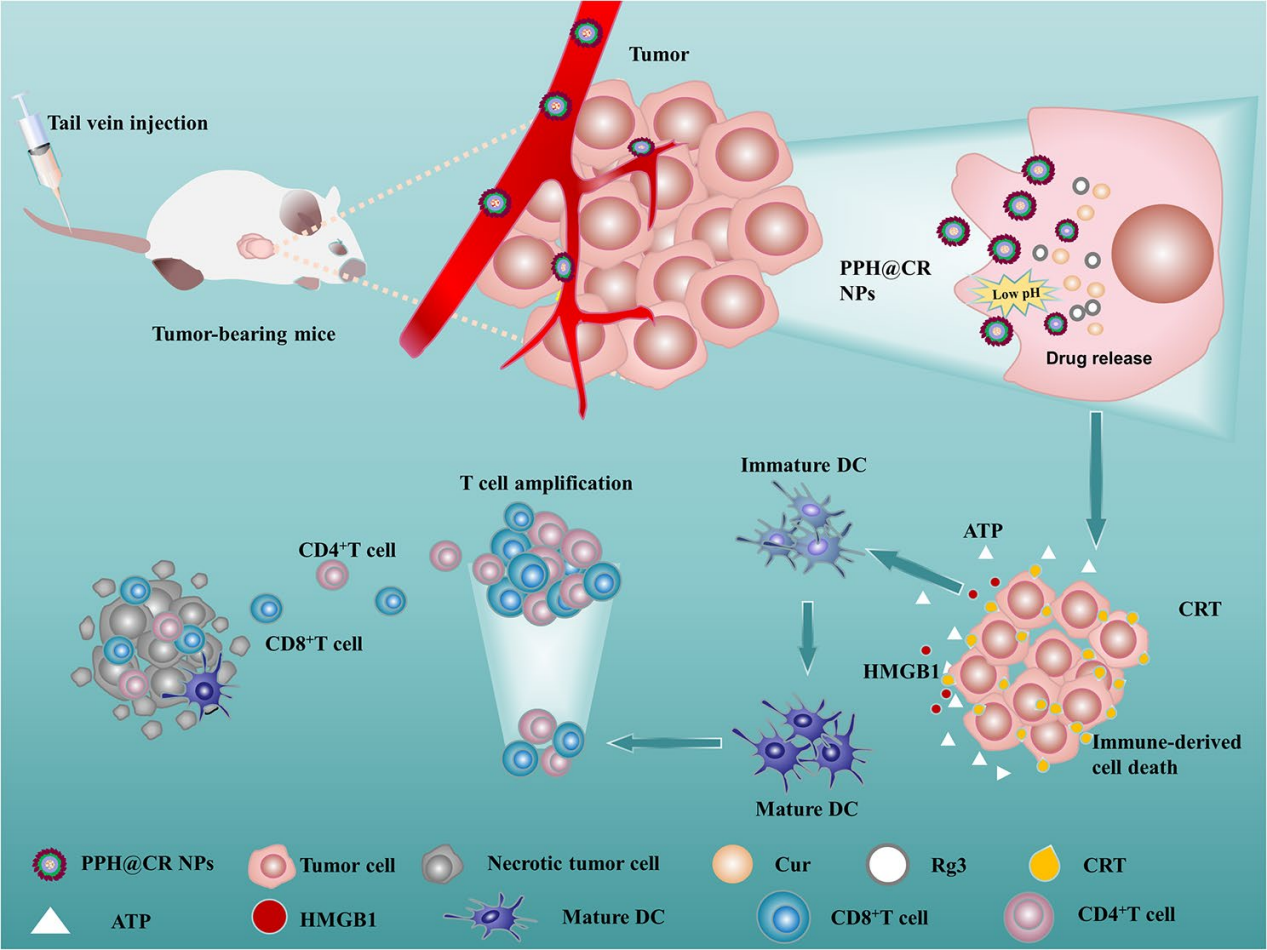
Schematic diagram of nanomicelles PPH@CR releasing drugs and inducing immunogenic cell death in tumor cells in response to low pH in the tumor microenvironment

In this study, we designed and synthesized the N-O zwitterionic polymer OPDEA-PGED by atom transfer radical polymerization (ATRP), which linked curcumin to the N-O zwitterionic polymer through vinyl ether bonding and encapsulated ginsenoside Rg3 to obtain hyperbranched zwitterionic drug-loaded micelles OPDEA-PGED-5HA@Cur@Rg3 (PPH@CR). Among them, curcumin was not only an anti-tumor drug but also a cross-linking agent for drug-loaded micelles, and this drug delivery system is expected to increase drug loading and have good stability, while having the characteristics of releasing drugs in response to tumor low pH. Not only that, PPH@CR may also reactivate the body’s anti-tumor immune response by inducing ICD and reducing the expression of PD-L1 in tumor cells, as well as help reduce tumor recurrence and treatment resistance by reducing cancer stem cells. Therefore, this study will provide a promising way of drug delivery for tumor treatment (Scheme. 1).

## Results

### Characterization of materials

In this study, we obtained PDEA-PGMA polymer through ATRP reaction, followed by ethylenediamine ring opening and hydrogen peroxide oxidation to obtain OPDEA-PGED, finally, the carrier material OPDEA-PGED-5HA was obtained by introducing alkyne groups at the amino terminus through amide bonds. The characterization results of PDEA-PGMA, PDEA-PGED, OPDEA-PGED and OPDEA-PGED-5HA are shown in Fig. S3.

### Characterization of empty micelle PPH as well as drug-loaded micelles PPH@C and PPH@CR

**Fig. 1:**
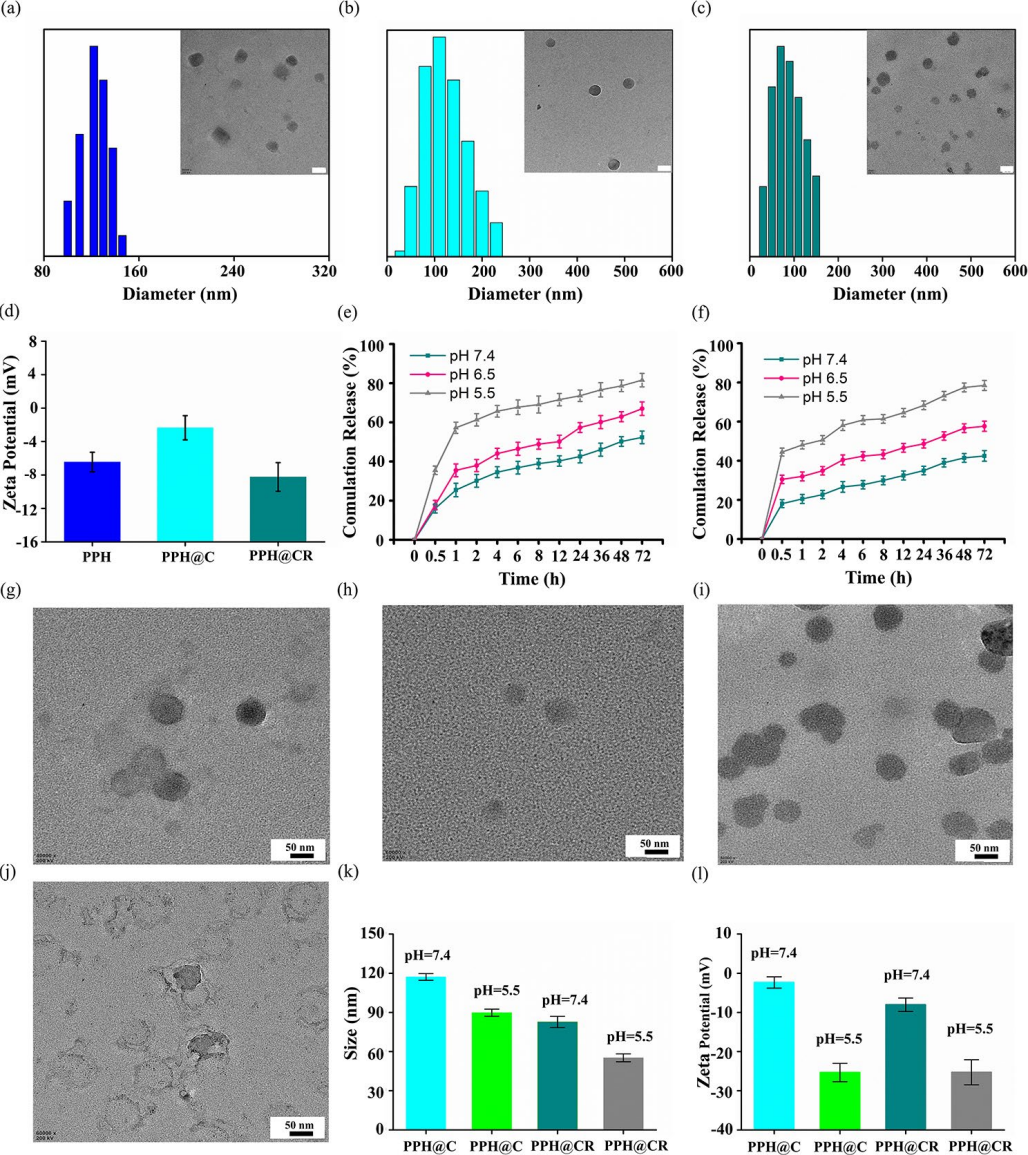
Characteristics of PPH, PPH@C, and PPH@CR. **(a-c)** The particle size distribution and TEM images of PPH (a), PPH@C (b) and PPH@CR (c) at room temperature. **(d)** The zeta potential diagrams of PPH, PPH@C, and PPH@CR at room temperature. **(e-f)** The drug release curves of Cur (e) and Rg3 (f) in PPH@CR at different pH environments. **(g-h)** The TEM images of PPH@C in saline at pH 7.4 (g) and pH 5.5 (h). **(i-j)** The TEM images of PPH@CR in saline at pH 7.4 (i) and pH 5.5 (j). **(k)** The particle size changes of PPH@C and PPH@CR in saline at different pH. **(l)** The zeta potential changes of PPH@C and PPH@CR in saline at different pH. All data are mean ± standard deviation (*n* = 3). (Scale bar, 50 nm)

The optimal particle size range for drug-loaded nanoparticles is 10–200 nm, and spherical nanoparticles are more likely to be endocytosed by tumor cells [18–20]. In this study, we obtained nanomicelles PPH@C and PPH@CR using the phenolic hydroxyl group in Cur to undergo a click reaction with alkyne, and the morphology of PPH, PPH@C and PPH@CR were observed using TEM. As shown in Fig. 1(a-c), the PPH, PPH@C and PPH@CR were spherical structures with average particle sizes of 85.16 ± 3.29 nm, 98.46 ± 2.43 nm, 69.72 ± 3.19 nm, respectively.

The particle size of the micelles was further detected using DLS, and the results showed that the average particle sizes of empty micelle PPH and drug-loaded micelles PPH@C and PPH@CR in aqueous solution were 103.46 ± 8.88 nm (Fig. 1a), 117.15 ± 2.55 nm (Fig. 1b) and 82.66 ± 4.27 nm (Fig. 1c), respectively. The particle size detected by DLS was significantly larger than that measured by TEM due to the different states of the micelles detected by DLS and TEM. In conclusion, the particle sizes of drug-loaded micelles PPH@C and PPH@CR were in the range of 10–200 nm, indicating that both can penetrate into the tumor through the tumor vascular system and avoid rapid renal clearance.

Zeta potential was further detected using a zeta potentiometer for empty and drug-loaded micelles. As shown in Fig. 1d, the average zeta potential of PPH, PPH@C, and PPH@CR were − 6.45 ± 1.17 mV, -2.35 ± 1.45 mV, and − 8.04 ± 1.72 mV, respectively, with nearly neutral potentials, which may also be related to the zwitterionic structure. In summary, PPH@C and PPH@CR have ideal zeta potential, size, and shape, which allow them to penetrate and remain in tumor tissues and facilitate endocytosis by tumor cells.

## Drug loading and stability of PPH@C and PPH@CR

In this study, Cur doubled as a crosslinker and antitumor component of nanomicelles PPH@C and PPH@CR. This drug-loaded micelle formed by the phenolic hydroxyl-alkyne click reaction was different from the traditional self-loading method, which greatly improved the stability and drug loading of the micelles and the formed vinyl ether bond can release drugs through acid hydrolysis [17]. We measured drug loading and encapsulation rate of PPH@C and PPH@CR with UV, and the drug loading of PPH@C was 14.47 ± 2.66%, and the encapsulation efficiency was 62.70 ± 11.55%. In the PPH@CR, the drug loading of Cur was 14.15 ± 2.33% and the encapsulation ratio was 61.4 ± 5.11%, and the drug loading of Rg3 was 2.26 ± 1.42% and the encapsulation ratio was 12.29 ± 2.64% (Table S1). The above results showed that the drug linked in the form of chemical bonds had a higher drug loading than the traditional physical method of encapsulating the drug.

The stability of PPH@C and PPH@CR were assessed by detecting changes in particle size and zeta potential of micelles at different time points. As shown in Fig. S2 and Table S2, the particle size of the PPH@C and PPH@CR did not change significantly after one month of storage in saline at 4 °C, which mainly because Cur was not only an antitumor drug but also acted as a cross-linker due to the presence of two phenolic hydroxyl groups in its structure. While the zeta potentials of PPH@C and PPH@CR did not change significantly within 30 days (Fig. S4 and Table S3), which attributed to the electroneutral nature of zwitterions [16]. These results showed that both PPH@C and PPH@CR have excellent stability as drug-loaded micelles.

### pH response of PPH@C and PPH@CR

The ideal drug delivery system should not only have excellent stability in the environment outside the target site but also have the ability to rapidly release the drug at the lesion site [21]. In this study, we simulated the in vivo environment to study the drug release of PPH@CR at different pH, the release of Cur (Fig. 1e) and Rg3 (Fig. 1f) in PPH@CR in saline at pH 7.4 were 52% and 42% within 72 h, respectively, and the release of Cur and Rg3 increased to 82% and 78% in saline at pH 5.5, respectively, these results showed that the cumulative release of Cur and Rg3 increased with the decreased of pH and obviously pH-dependent. We further observed the morphological changes of PPH@C and PPH@CR in saline at pH 7.4 and pH 5.5 using TEM, the PPH@C (Fig. 1g) and PPH@CR (Fig. 1i) had a homogeneous spherical structure with well-defined boundaries in saline at pH 7.4, while the same features were not obvious at pH 5.5, showing blurred and unclear boundaries and irregular morphology (Fig. 1h, j). The particle size and zeta potential changes were further detected using DLS, and the results showed that the particle sizes of PPH@C and PPH@CR were 117.15 ± 2.55 nm and 82.66 ± 4.27 nm in saline at pH 7.4, and decreased to 105.31 ± 3.20 nm and 55.21 ± 3.01 nm in saline at pH 5.5, respectively (Fig. 1k and Table S4). In addition, the zeta potentials of PPH@C and PPH@CR in saline at pH 7.4 were − 2.35 ± 1.45 mV and − 8.04 ± 1.72 mV, respectively, while the zeta potentials in saline at pH 5.5 were − 25.34 ± 2.35 mV and − 25.26 ± 3.20 mV, respectively (Fig. 1l and Table S4). These results indicated that the morphology, particle size and zeta potential of the nanomicelles PPH@C and PPH@CR change with the decrease of pH, which supported that the characteristics of the nanomicelle in response to low pH to release the drugs.

### In vitro cytotoxicity

**Fig. 2:**
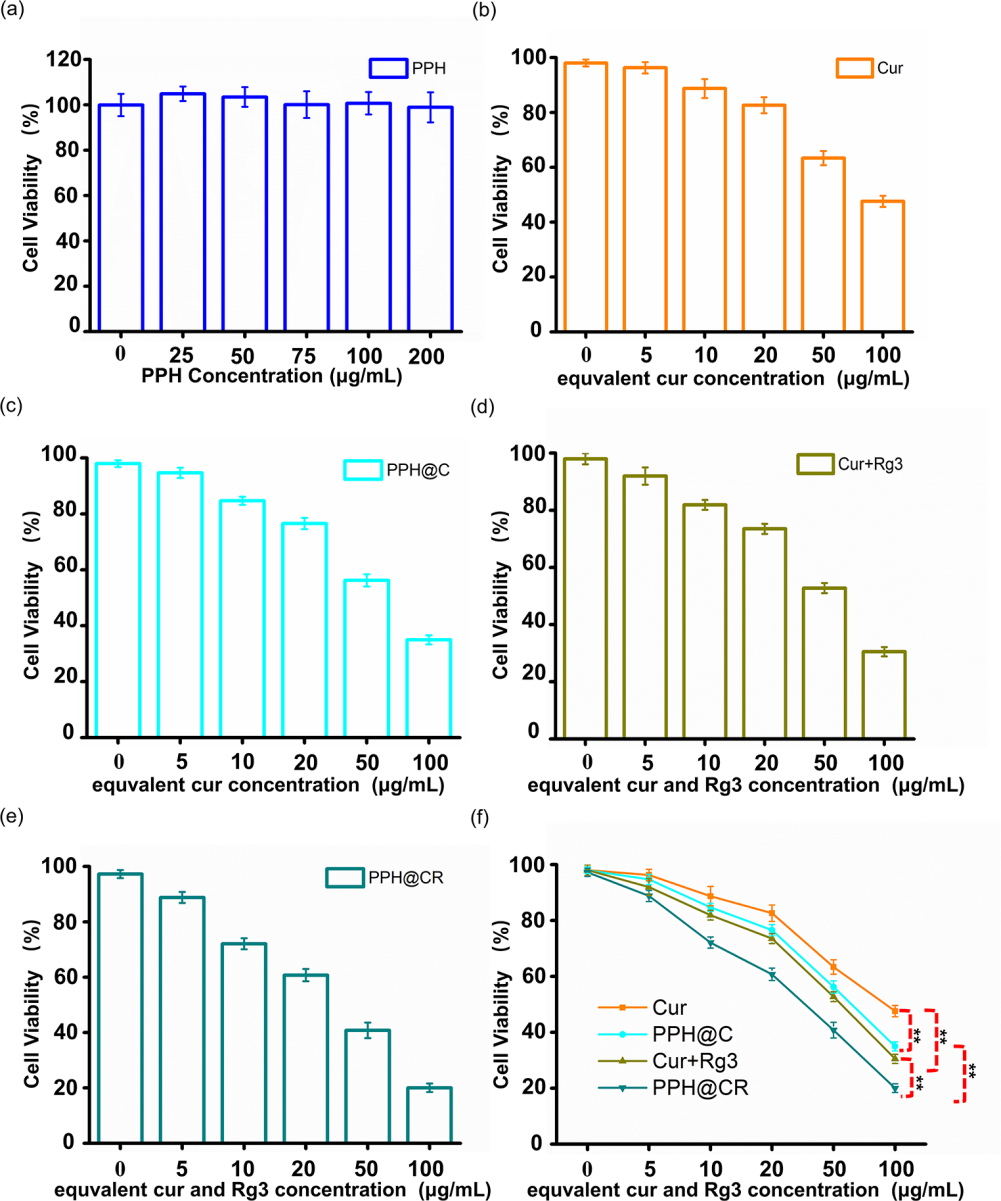
In vitro cytotoxicity of free drugs and nanomicelles. **(a)**In vitro cytotoxicity of different concentrations of PPH acting on 4T1 cells for 24 h. **(b)**In vitro cytotoxicity of different concentrations of Cur acting on 4T1 cells for 24 h. **(c)**In vitro cytotoxicity of different concentrations of drug-loaded micelle PPH@C acting on 4T1 cells for 24 h. **(d)**In vitro cytotoxicity of different concentrations of Cur + Rg3 acting on 4T1 cells for 24 h. **(e)**In vitro cytotoxicity of different concentrations of drug-loaded micelle PPH@CR acting on 4T1 cells for 24 h. **(f)** Comparison of in vitro cytotoxicity of different concentrations of each group of drugs acting on 4T1 cells for 24 h. Data are presented as mean ± standard deviation (*n* = 3), ** *p* < 0.01

The ideal drug delivery material is based on low cytotoxicity, so we tested the effect of nanomicelles on the proliferative activity of 4T1 cells using the CCK-8 kit. As shown in Fig. 2a, the cell viability was still higher than 90% when the PPH concentration up to 200 µg/mL and showing good cytocompatibility. The toxicity of nanomicelles PPH@C and PPH@CR to 4T1 cells was further detected with CCK-8 kit, and the results showed that the drugs in each group could inhibit the proliferation of 4T1 cells in a concentration-dependent manner (Fig. 2b-f), and the PPH@CR group had the strongest cytotoxicity with IC_50_ value of 29.94 µg/mL, followed by Cur + Rg3, PPH@C and free Cur group with IC_50_ of 49.54 µg/mL, 57.35 µg/mL and 77.67 µg/mL, respectively. The above results showed that the nanomicelles group had stronger cytotoxicity on 4T1 cells compared to the free drug group at the same concentration, this may be related to the different uptake mechanisms of nanomedicines and free Cur by tumor cells, and also indicated that PPH@C and PPH@CR can be effectively internalized by tumor cells to produce cytotoxicity.

### In vitro cell uptake

**Fig. 3:**
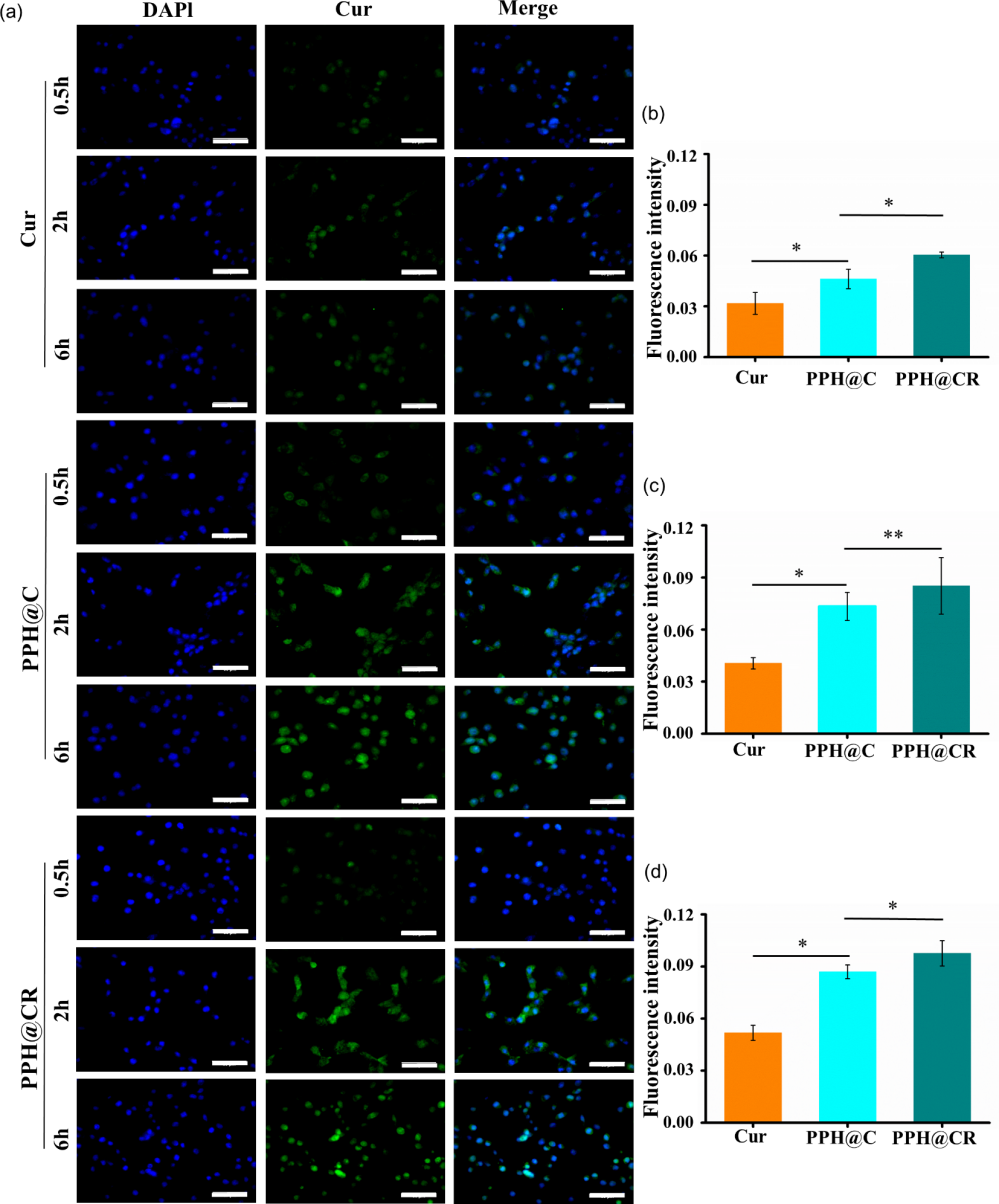
The uptake of Cur, PPH@C, and PPH@CR by 4T1 cells. **(a)** The fluorescence microscopy images of 4T1 cells incubated with Cur, PPH@C, and PPH@CR for 0.5 h, 2 h, and 6 h, respectively (Scale bar, 50 μm). **(b)** The fluorescence intensity of 4T1 cells incubated with Cur, PPH@C, and PPH@CR for 0.5 h was quantified with ImageJ. **(c)** The fluorescence intensity of Cur in 4T1 cells incubated with Cur, PPH@C, and PPH@CR for 2 h was quantified with ImageJ. **(d)** The fluorescence intensity within 4T1 cells incubated with Cur, PPH@C, and PPH@CR for 6 h was quantified with ImageJ. Data are presented as mean ± standard deviation (*n* = 3), *p < 0.05, **p < 0.01

Drug loaded nanoparticles can only be effectively internalized by target cells to fully exert therapeutic effects. In this study, we observed the uptake of Cur, PPH@C, and PPH@CR by 4T1 cells using CLSM. As shown in Fig. 3a, the free drug Cur, nanomicelles PPH@C and PPH@CR could be taken up by 4T1 cells, and the fluorescence signal gradually increased with the extension of incubation time, indicating that the uptake of drugs by 4T1 cells was time-dependent (where blue fluorescence represents nucleus, and green fluorescence represents Cur or Cur-containing nanomedicine). And the fluorescence intensity of Cur in 4T1 cells was further quantified with ImageJ, the results showed that there was a difference in the uptake of Cur, PPH@C, and PPH@CR by 4T1 cells (Fig. 3b-d), among which PPH@CR was most uptaken by 4T1 at the same time, followed by PPH@C and Cur, which may be related to the fact that the particle size of the PPH@CR was less than PPH@C. In addition, the uptake of nanomicelle PPH@C by 4T1 cells was more than that of free drugs, indicating that nanomicelles are more easily uptaken by 4T1 cells.

### Uptake mechanism

**Fig. 4:**
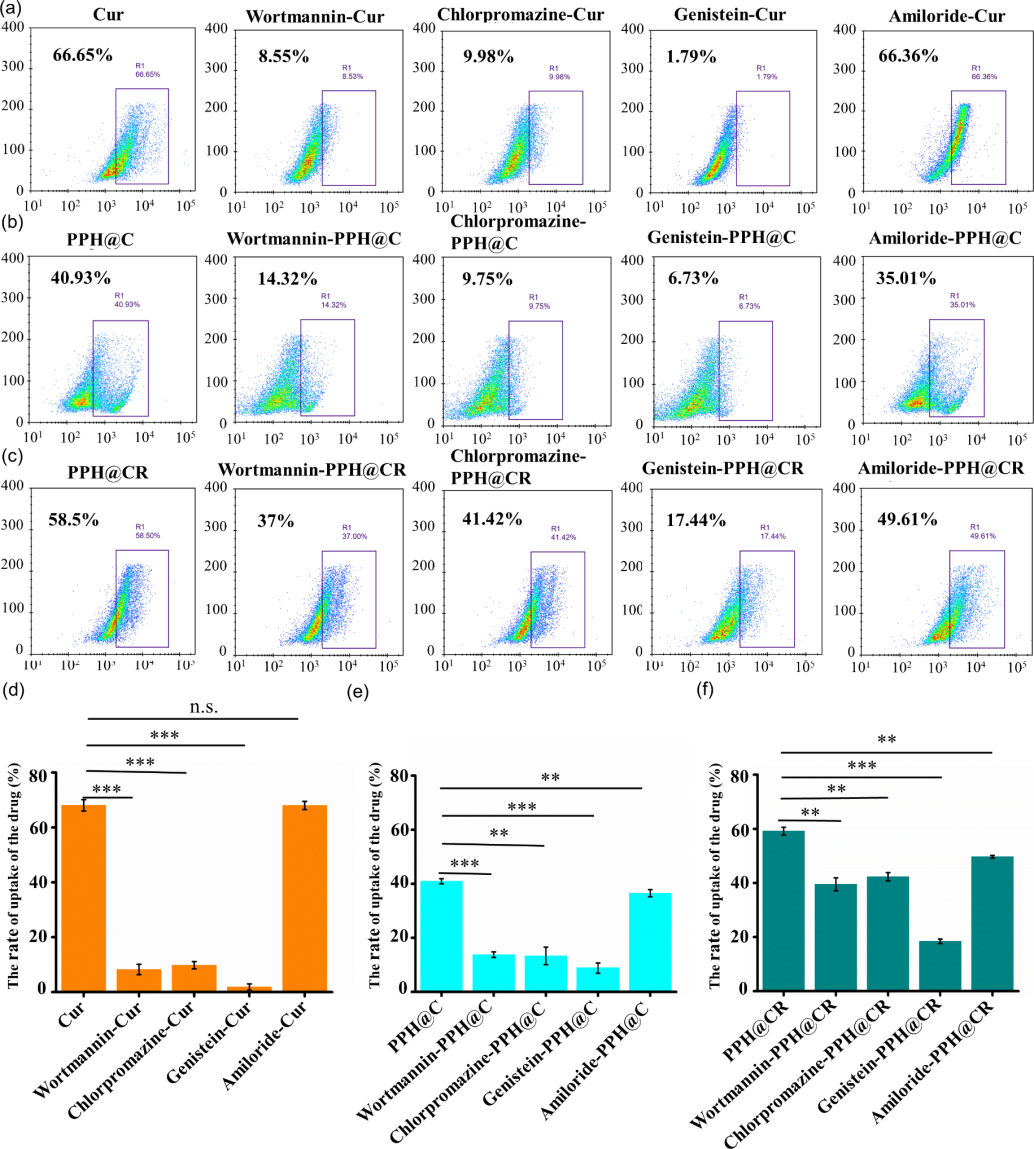
The mechanism of uptake of Cur, PPH@C and PPH@CR by 4T1 cells. **(a)** The flow cytometry diagrams of Cur uptake by 4T1 cells pretreated with different endocytic inhibitors. **(b)** The flow cytometry diagrams of PPH@C uptake by 4T1 cells pretreated with different endocytic inhibitors. **(c)** The flow cytometry diagrams of PPH@CR uptake by 4T1 cells pretreated with different endocytosis inhibitors. **(d-f)** The comparison diagrams of flow cytometry analysis of Cur (d), PPH@C (e), and PPH@CR (f) uptake by 4T1 cells pretreated with different endocytosis inhibitors. Data are presented as mean ± standard deviation (*n* = 3), NS, not significant, ** *p* < 0.01, *** *p* < 0.001

Improving drug penetration has become the primary task of improving the therapeutic effect of nanomedicines, and transcytosis has attracted attention as a potential solution to address the limitations associated with passive drug delivery, among which clathrin-mediated endocytosis, caveolae-mediated endocytosis and macropinocytosis are the most relevant to transcytosis [22]. In this study, the 4T1 cells were pretreated with endocytosis inhibitors 5-(N, N-Hexamethylene)-amiloride (micropinocytosis inhibitor), genistein (caveolae-mediated endocytosis inhibitor), chlorpromazine (clathrin-mediated inhibitor of endocytosis) and wortmann penicillin (macropinocytosis inhibitor), respectively, and their uptake were analyzed using flow cytometry in order to explore the uptake mechanism of nanomicelles PPH@C and PPH@CR by 4T1 cells. As shown in Fig. 4(a-c), compared with 4T1 cells pretreated without inhibitors, the uptake of Cur (8.55%), PPH@C (14.32%) and PPH@CR (37%) were significantly reduced in tumor cells pretreated with womannpenicillin, chlorpromazine and genistein, and the uptake of Cur (66.36%) was not effected by tumor cells pretreated with 5-(N, N-Hexamethylene)-amiloride, but the uptake of PPH@C (35.01%) and PPH@CR (49.61%) decreased. These results indicated that the main pathways for tumor cells to take up Cur, PPH@C, and PPH@CR were caveolae-mediated endocytosis, clathrin-mediated endocytosis, and macropinocytosis. In addition, PPH@C and PPH@CR could also be taken up by 4T1 cells through the micropinocytosis pathway (Fig. 4d-f), which indicateed that Cur, PPH@C and PPH@CR can not only be rapidly endocytosed by tumor cells, but also closely related to transcytosis, which is conducive to the penetration of drugs into the deep tumor, thereby improving the drug penetration rate and giving full play to the anti-tumor efficacy [22].

### PPH@C and PPH@CR induce ICD in tumor cells

**Fig. 5:**
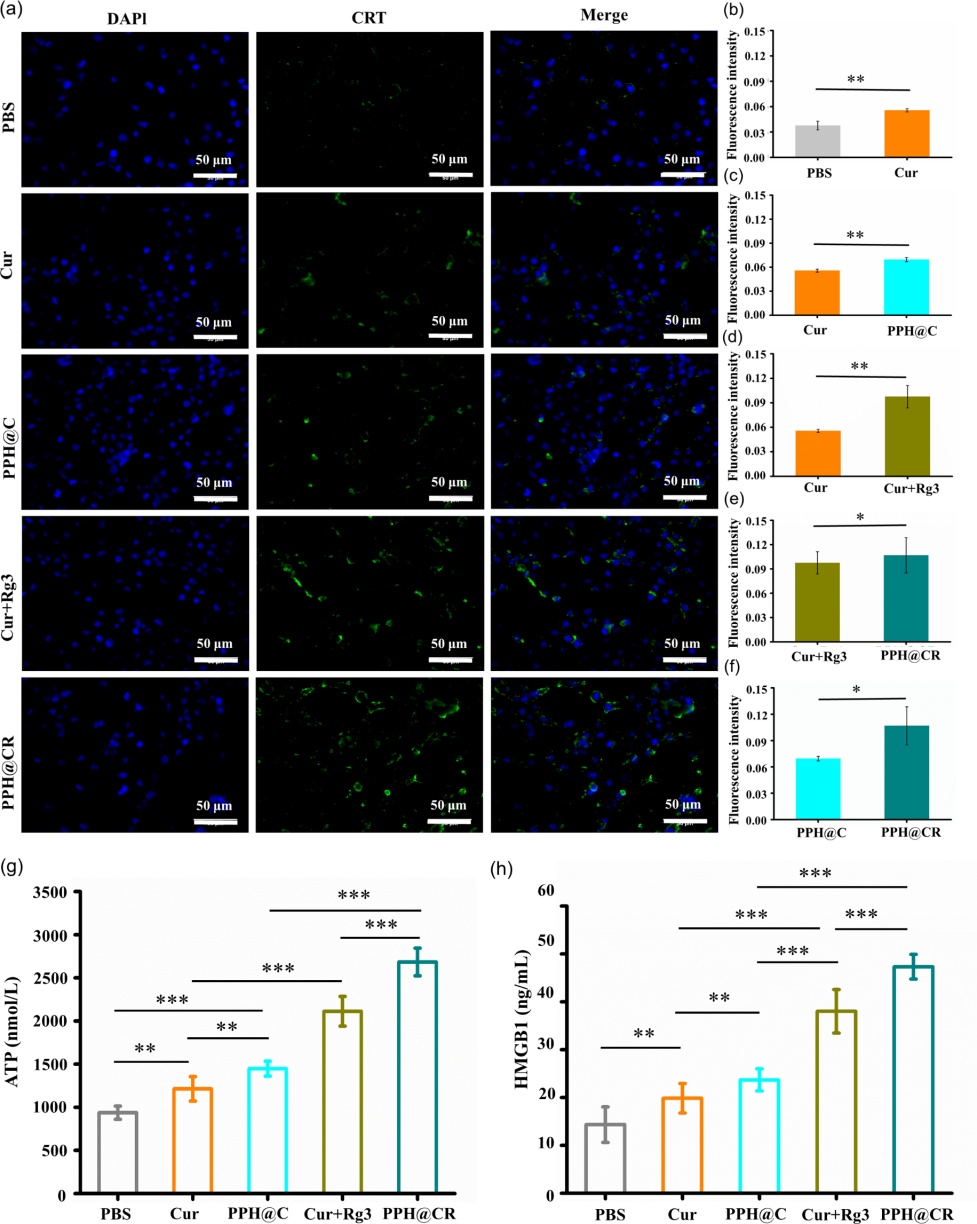
The immunogenic cell death induced by free drugs and nanomicelles in 4T1 cells. **(a)** The immunofluorescence staining of CRT in 4T1 cells incubated with PBS, Cur, PPH@C, Cur + Rg3 and PPH@CR for 24 h, respectively (Scale bar, 50 μm). **(b-f)** The immunofluorescence intensity of CRT in each group was quantified with ImageJ after 24 h of incubation with 4T1 cells. **(g)** The levels of ATP in the supernatant of 4T1 cells after 24 h incubation with PBS, Cur, PPH@C, Cur + Rg3, and PPH@CR, respectively. **(h)** The levels of HMBG1 in the supernatant of 4T1 cells after 24 h incubation with PBS, Cur, PPH@C, Cur + Rg3, PPH@CR, respectively. Data are presented as mean ± standard deviation (*n* = 3), *p < 0.05, **p < 0.01, ***p < 0.001

Immunogenic cell death is a rare form of immunostimulatory cell death that can reactivate tumor-specific immune responses and is characterized by the release of DAMPs, including exposure to CRT on the cell surface and release of ATP and HMGB1 [23]. Studies have shown that both Cur and Rg3 can induce ICD in tumor cells but the effect of single agent inducing ICD in cells is weak [24], while nanodrug delivery systems can deliver multiple ICD inducers simultaneously to increase ICD effect [25] In this experiment, we evaluated the PPH@CR-induced ICD in tumor cells by measuring CRT exposure and ATP and HMGB1 release after drug action on 4T1 cells. The results are shown in Fig. 5a, and the fluorescence intensity of CRT in the five dosing groups from strong to weak was PPH@CR, Cur + Rg3, PPH@C, Cur and PBS group, respectively. Among them, the PPH@CR group had the strongest effect on inducing CRT exposure in 4T1 cells and was significantly higher than that in other groups (Fig. 5b-f). And the levels of ATP and HMGB1 in the supernatant of 4T1 cells were further detected by mouse ATP ELISA kit and HMGB1 ELISA kit after 24 h of drug action in each group. The results were consistent with the results of CRT and the release of ATP release in descending order was PPH@CR (2684.14 nmol/L), Cur + Rg3 (2112.19 nmol/L), PPH@C (1447.75 nmol/L), Cur (1213.58 nmol/L), and PBS (937.19 nmol/L) group, among which the PPH@CR group had the highest ATP content, which was 27.08% and 85.40% higher than that of the Cur + Rg3 and PPH@C group, respectively (Fig. 5g). The release of HMGB1 from high to low was PPH@CR (47.33 ng/mL), Cur + Rg3 (38.02 ng/mL), PPH@C (23.68 ng/mL), Cur (19.86 ng/mL) and PBS (14.33 ng/mL), respectively, and the PPH@CR group released the largest amount of HMGB1, which was 24.49% and 99.87% higher than that in the Cur + Rg3 group and the PPH@C group, respectively (Fig. 5h). The above experimental results showed that nanomicelles PPH@C and PPH@CR could induce ICD in 4T1 tumor cells and this effect was better than that of free drugs, and the PPH@CR loaded with two drugs had the most significant effect on inducing ICD in tumor cells. At the same time, we can also see that Rg3 could enhance the ability of Cur to induce ICD in tumor cells in both the free drug group and the nanodrug group.

### PPH@C and PPH@CR promote DCs maturation and reduce PD-L1 in 4T1 cells

**Fig. 6:**
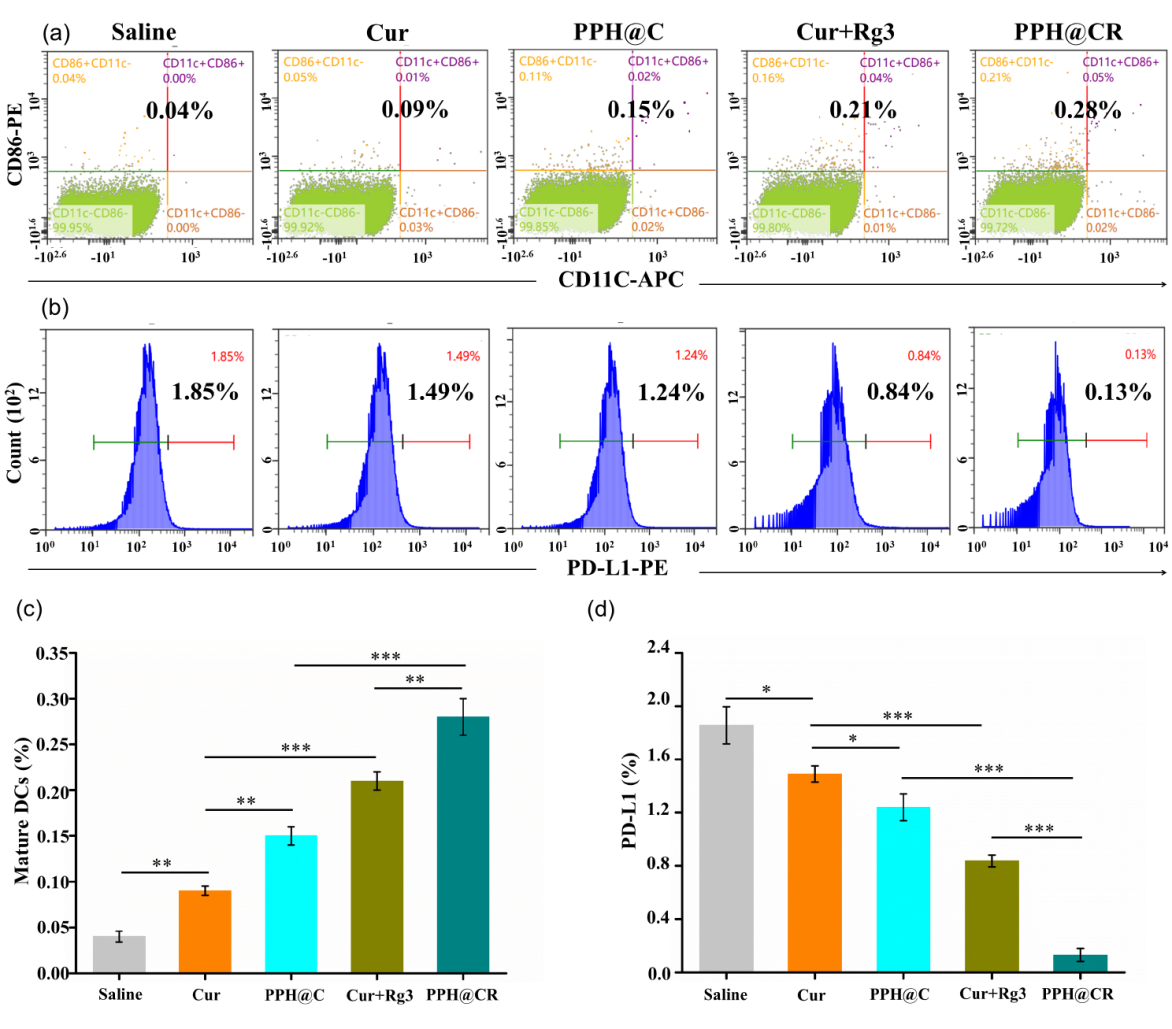
Free drugs and nanomicelles promote DCs maturation and reduce PD-L1 in tumor cells in vitro. **(a)** Flow cytometry profiles of CD11c^+^/CD86^+^/CD11c^+^CD86^+^ cell populations (markers of mature dendritic cells) after drug action on 4T1 cells in vitro (saline, Cur, PPH@C, Cur + Rg3, and PPH@CR group). **(b)** Flow cytometry profile of PD-L1 cell population after each group (saline, Cur, PPH@C, Cur + Rg3, and PPH@CR group) of drugs acted on 4T1 cells in vitro. **(c)** Comparison chart of DCs maturation after drug action on 4T1 cells in each group. **(d)** Comparison chart of PD-L1^+^ 4T1 cells after drug action on 4T1 cells in each group. Data are presented as mean ± standard deviation (*n* = 3), *p < 0.05, **p < 0.01, ***p < 0.001

Mature DCs have antigen-presenting properties that can elicit an immune response. We analyzed the number of mature DCs in the co-culture system by flow cytometry, and the results showed that the PPH@CR group had the largest number of mature DCs, followed by Cur + Rg3, PPH@C, Cur, and PBS group, indicating that the drugs in each group could promote the maturation of DCs compared with the PBS group, and the PPH@CR effect was the strongest (Fig. 6). In addition, studies have shown that both Cur and Rg3 can reduce the expression of PD-L1 on the surface of tumor cells [26–28]. In this study, the number of PD-L1^+^ cells in each group was analyzed by flow cytometry after 48 h of drug action on 4T1 cells, and the results showed that the drugs in each group (Cur, PPH@C, Cur + Rg3 and PPH@CR group) could reduce the number of PD-L1^+^ 4T1 cells compared with the PBS group, and the number of PD-L1^+^ 4T1 cells from less to more were PPH@CR, Cur + Rg3, PPH@C, Cur, and PBS group. Among them, the number of PD-L1^+^ 4T1 cells in the PPH@CR group was the lowest, indicating that PD-L1^+^ 4T1 cells could be significantly reduced PPH@CR in vitro (Fig. 6).

### PPH@C and PPH@CR inhibit tumor cell migration

**Fig. 7:**
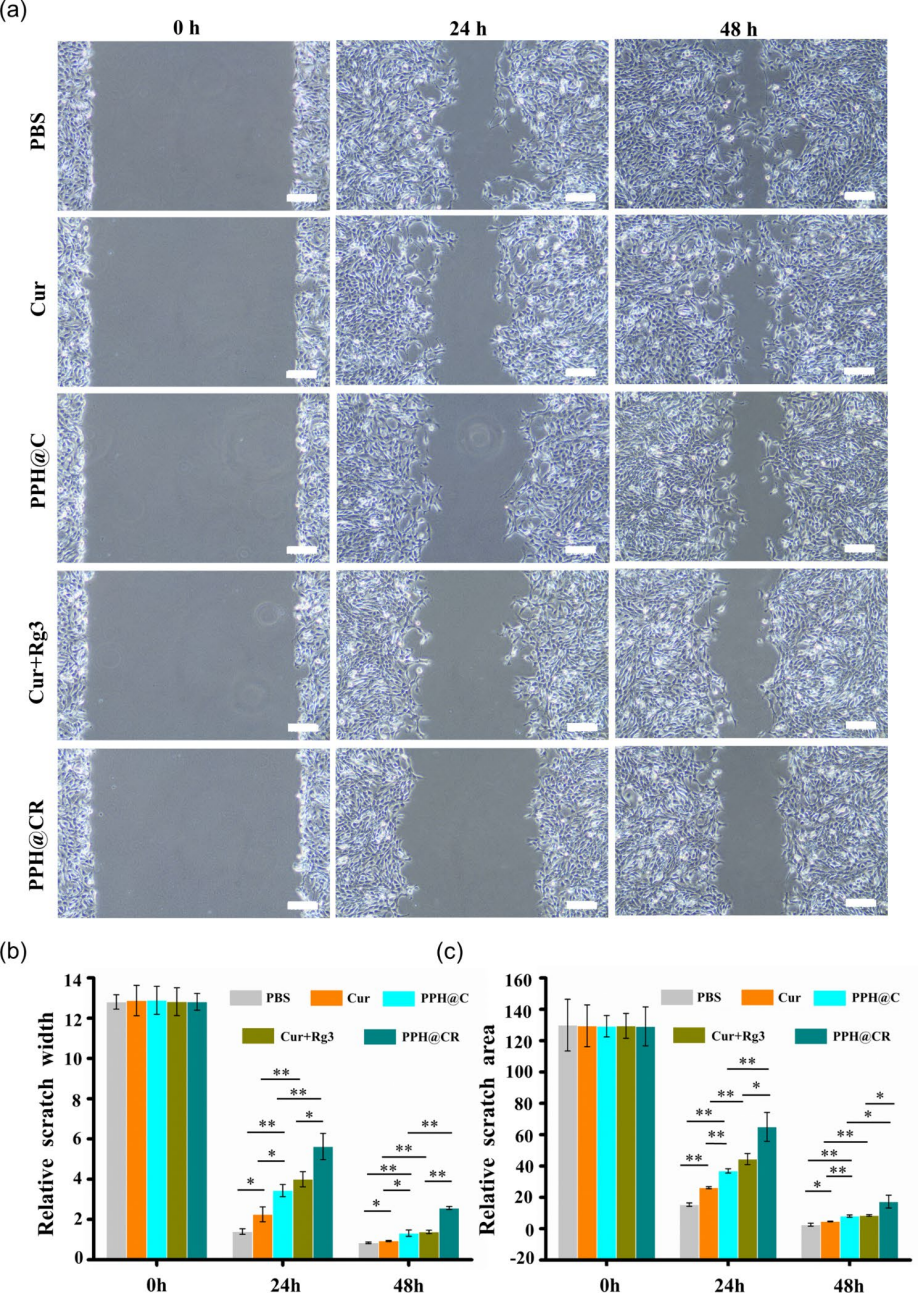
The inhibition of tumor cell migration by free drugs and nanomicelles. **(a)** The microscopic images of PBS, Cur, PPH@C, Cur + Rg3, PPH@CR acting on 4T1 cells for 24 h and 48 h (Scale bar, 100 μm). **(b)** The relative scratch widths of each group of drugs acting on 4T1 cells for 24 h and 48 h. **(c)** The relative scratch area of each group of drugs acting on 4T1 cells for 24 h and 48 h. Data are presented as mean ± standard deviation (*n* = 3), *p < 0.05, **p < 0.01

Tumor metastasis is the leading cause of cancer-related death and reducing the risk of cancer metastasis is the key to treating cancer and improving patient survival, especially for TNBC with strong invasiveness and high distant metastasis rate [29, 30]. In this study, the effects of PPH@C and PPH@CR on the migration of 4T1 cells were evaluated by scratch test, and the results are shown in Fig. 7a, the drugs in each group could inhibit 4T1 cell migration compared with the control group, and the cell migration rates in each group was 89.07% (PBS), 82.22% (Cur), 73.34% (PPH@C), 68.83% (Cur + Rg3) and 56.11% (PPH@CR) after 24 h of treatment, and 93.45% (PBS), 92.52% (Cur), 90.28% (PPH@C), 89.23% (Cur + Rg3) and 79.94% after 48 h of treatment (PPH@CR) (Fig. 7b), of which the PPH@CR group had the greatest inhibitory effect on cell migration. These results indicated that PPH@C and PPH@CR have a strong inhibitory effect on tumor cell migration (Fig. 7b, c).

### In vivo anti-tumor efficacy

**Fig. 8:**
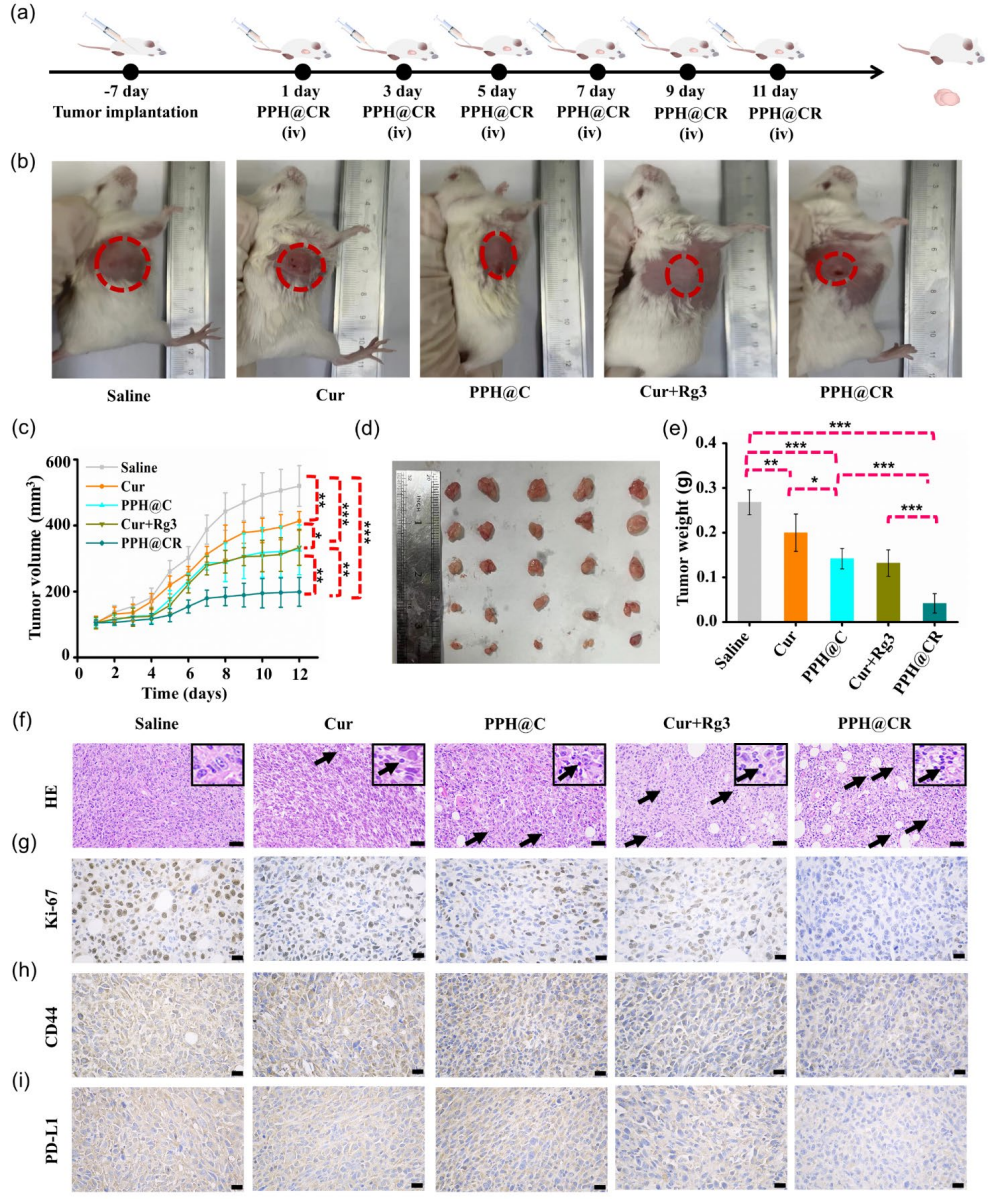
In vivo dosing regimen and tumor suppression effects of free drugs and nanomicelles. **(a)** Dosing time (day 1, 3, 5, 7, 9, 11) and mode of administration (tail vein injection) of female BALB/c tumor-bearing mice. **(b)** The tumor growth in each group (saline, Cur, PPH@C, Cur + Rg3 and PPH@CR group) on the 12th day after the start of treatment. **(c)** The average tumor growth curves of tumor-bearing mice in each group. **(d)** The anatomical images of tumors on day 12 after initiation of treatment in tumor-bearing mice in each group. **(e)** The average tumor weight on day 12 after initiation of treatment in each group of tumor-bearing mice. **(f)** The H&E staining of tumor tissues of tumor-bearing mice in each group after treatment (Scale bar, 50 μm). **(g-i)** The immunohistochemistry of tumor tissues of tumor-bearing mice in each group after treatment (Scale bar, 20 μm). Data are presented as mean ± standard deviation (*n* = 3), *p < 0.05, **p < 0.01, ***p < 0.001

The in vivo efficacy of drug-loaded nanoparticles is the most important indicator to evaluate the therapeutic effect of nanomedicines. In this study, we evaluated the antitumor activity of PPH@CR in mice by 4T1 orthotopic tumor models. The dosing regimen is shown in Fig. 8a, where tumors grew rapidly in the saline group, and the tumor volume in the other groups was smaller than that in the saline group, with the smallest volume being the PPH@CR group (Fig. 8b-c). At the end of the experiment, the tumors of mice in each group are shown in Fig. 8d-e, in which the average tumor weight of the PPH@CR group was significantly lower than that of the other groups, and the tumor suppression rate was 84.32%, followed by the Cur + Rg3 (50.75%), PPH@C (47.01%) and Cur (35.37%) group, indicating that PPH@CR could significantly inhibit tumor growth, and the PPH@CR loaded with double drugs had the most significant tumor inhibitory effect compared with the free drug and nanomicellar PPH@C.

We further evaluated the therapeutic effect of each group of drugs by tumor tissue morphology and the results are shown in Fig. 8f. Mice tumor tissues treated with saline consisted of tightly packed cells with typical cancer cell morphology, while the tumor cells in the other groups showed different degrees of cell necrosis such as nuclear condensation and nuclear lysis, among which a small number of broken and condensed nuclei could be observed in the Cur and PPH@C group, and this phenomenon was more significant in the Cur + Rg3 and PPH@CR group, among which PPH@CR was the most significant (where the black arrows in the Cur, PPH@C, Cur + Rg3, PPH@CR groups refer to the necrotic tumor cells), indicating that PPH@CR had the strongest effect on tumor necrosis. These results can be attributed to the fact that nanomedicines PPH@C and PPH@CR have the property of responding to the low pH of the tumor microenvironment, which allowed the loaded drug to target tumor release thereby leading more necrosis at the cellular level.

Ki-67 is a nuclear proliferative antigen and its high expression generally indicates active cell proliferation [31]. In this study, we detected the expression of Ki-67 in tumor tissues of tumor bearing mice treated with various drugs and the results are shown in Fig. 8g, the expression of Ki-67 was the most significant in the saline treatment group, followed by Cur, PPH@C, Cur + Rg3 and PPH@CR group, among which the PPH@CR group had the least expression of Ki-67, indicating that the tumor cells of tumor-bearing mice after PPH@CR treatment had the weakest proliferation ability.

### PPH@C and PPH@CR reduce stem cells in tumor tissues and reduce tumor PD-L1 expression

Cancer stem cells are the main cause of tumor recurrence and resistance to treatment [32], and CD44 is the most commonly used molecular marker for identifying TNBC stem cells and numerous studies have demonstrated that the expression of CD44 is associated with tumor grade, tumor recurrence, and metastasis in breast cancer patients [7, 33]. In this study, we detected the expression of CD44 in tumor tissues of tumor bearing mice treated with various drugs using immunohistochemistry. As shown in Fig. 8h, the expression of CD44 in tumor tissue from most to least was as follows: saline, Cur, PPH@C, Cur + Rg3 and PPH@CR group, and among which the CD44 expression of tumor tissue in the PPH@CR group was the least and significantly lower than that in the other groups, indicating that PPH@CR had the strongest ability to reduce stem cells in tumor tissues, which helps to reduce tumor recurrence and resistance to treatment.

PD-L1 is an immune checkpoint protein that is highly expressed in a variety of cancers including breast cancer, and which can promote the immune escape of cancer cells by binding to PD-1 on T cells [5] In this study, we measured the expression of PD-L1 in tumor cells after drug treatment in each group by immunohistochemistry. As shown in Fig. 8i, the least expression of PD-L1 was in the PPH@CR group, suggesting that PPH@CR helps to reduce tumor immune escape.

### PPH@C and PPH@CR induce ICD in tumor cells in vivo

**Fig. 9:**
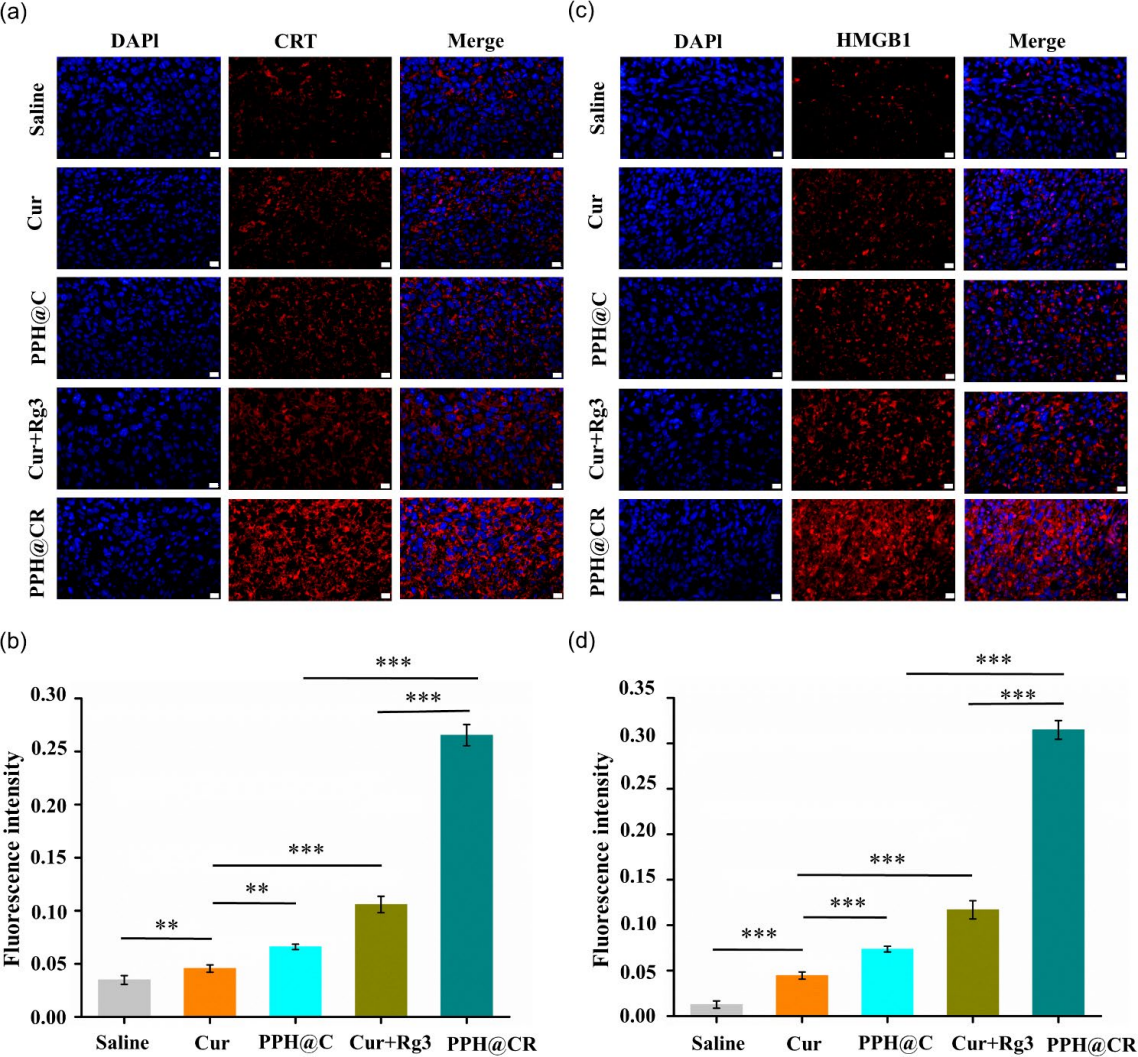
The immunogenic cell death induced by free drugs and nanomicelles in vivo. **(a)** The immunofluorescence staining of CRT in mice tumor tissues after drug treatment in each group (saline, Cur, PPH@C, Cur + Rg3, PPH@CR). **(b)** The fluorescence intensity of (a) was quantified with ImageJ. **(c)** The immunofluorescence staining of HMBG1 in mice tumor tissues after drug treatment in each group (saline, Cur, PPH@C, Cur + Rg3, PPH@CR). **(d)** The fluorescence intensity of (c) was quantified with ImageJ. (Scale bar, 20 μm). Data are presented as mean ± standard deviation (*n* = 3), **p < 0.01, ***p < 0.001

It has been confirmed that PPH@C and PPH@CR can induce ICD in 4T1 tumor cells in vitro cell experiments, and we further detected the expression of CRT in tumor tissues by immunofluorescence. As shown in Fig. 9a, only a small amount of CRT fluorescence was detected in the tumor tissues of the tumor-bearing mice treated with saline, while a more significant CRT fluorescence signals were detected in the tumor tissues of the other groups. Further quantitative analysis of the fluorescence intensity of CRT in tumor sections with ImageJ and the results are shown in Fig. 9b, the average fluorescence intensity of CRT in each group was 0.035 (saline group), 0.0452 (Cur group), 0.0686 (PPH@C group), 0.0995 (Cur + Rg3 group) and 0.275 (PPH@CR group), among which the PPH@CR group had the highest CRT fluorescence intensity.

The expression of HMGB1 in tumor tissues after drug treatment in each group was further detected by immunofluorescence. As shown in Fig. 9c, the fluorescence of HMGB1 in tumor tissues after saline treatment basically overlapped with the nucleus, which was due to the fact that HMGB1 is mainly expressed in the nucleus in normal cells, while HMGB1 in 4T1 cells will be transferred from the nucleus to the cytoplasm through the nuclear membrane after being treated with the drug and then released into the extracellular space through the plasma membrane [34]. We further quantified the fluorescence intensity of HMGB1 in tumor sections, and the results showed that the fluorescence intensity of each group was 0.013 (saline group), 0.045 (Cur group), 0.073 (PPH@C group), 0.117 (Cur + Rg3 group) and 0.315 (PPH@CR group), and among which the highest expression of HMGB1 outside the nucleus was in the PPH@CR group (Fig. 9d). In summary, the PPH@C and PPH@CR could not only induce ICD in tumor cells in vitro but also in vivo, and this induction effect was higher than that of the free drug group, indicating that nanomicelles can enrich tumor tissues and enhance ICD.

### PPH@C and PPH@CR increase the proportion of mature DCs in vivo

**Fig. 10:**
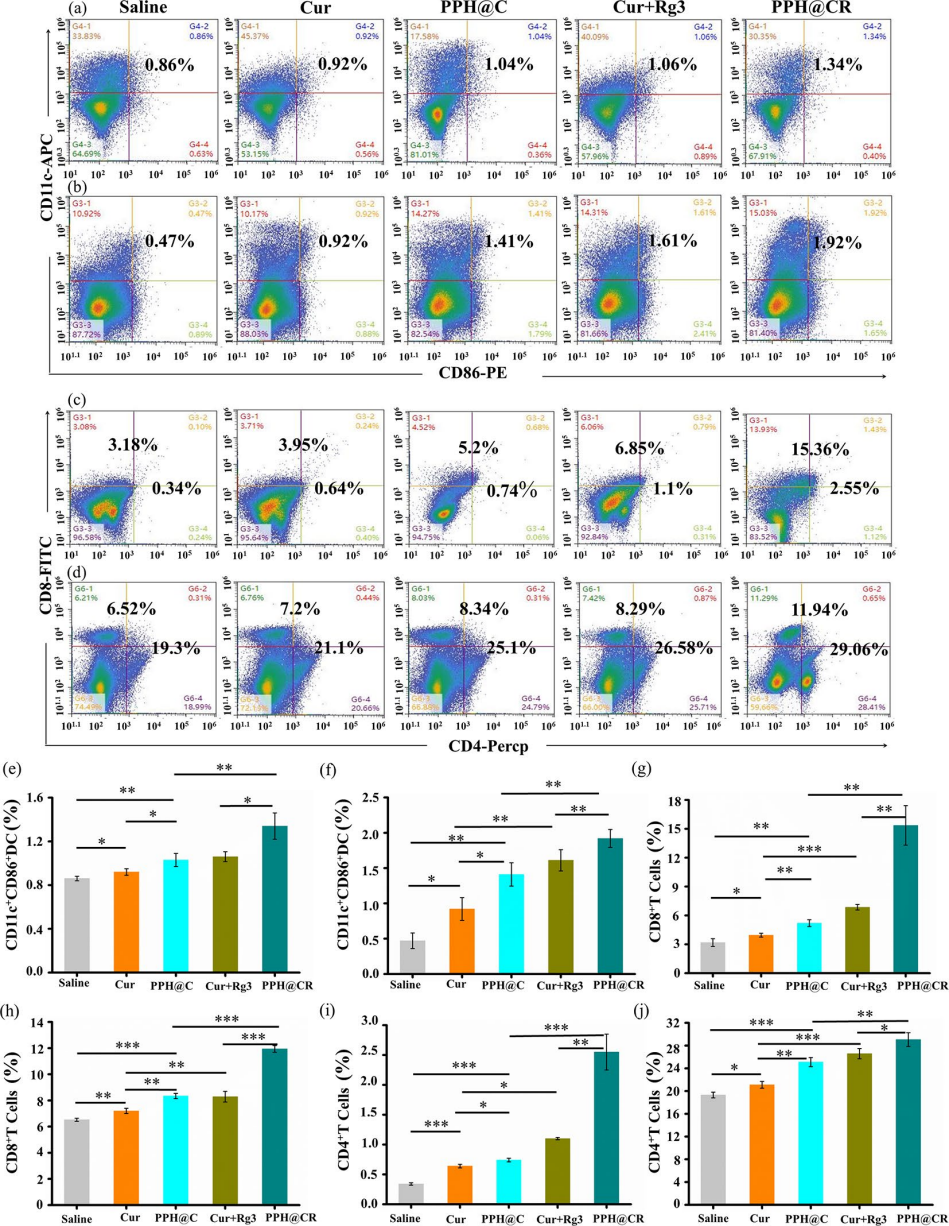
Free drugs and nanomicelles promote DCs maturation and effector T cells proliferation in mice. **(a)** Flow cytometry profiles of CD11c^+^CD86^+^ cell populations (markers of mature dendritic cells) in tumor tissues of tumor-bearing mice in each group (saline, Cur, PPH@C, Cur + Rg3 and PPH@CR group) on day 12 after initiation of treatment. **(b)** Flow cytometry profile of CD11c^+^CD86^+^ cell population in spleen tissues of tumor-bearing mice in each group on day 12 after initiation of treatment. **(c)** Flow cytometry profiles of CD4^+^ T cell populations and CD8^+^ T cell populations in tumor tissues of tumor-bearing mice in each group on day 12 after initiation of treatment. **(d)** Flow cytometry profiles of CD4^+^ T cell populations and CD8^+^ T cell populations in spleen tissues of tumor-bearing mice on the 12th day after initiation of treatment. **(e**,** f)** Comparison of CD11c^+^CD86^+^ cells in tumor tissues (e) and spleen tissues (f) of tumor-bearing mice in each group on the 12th day after starting treatment. **(g**,** h)** Comparison of CD8^+^ T cells in tumor tissue (g) and spleen tissue (h) on the 12th day after initiation of treatment in each group of tumor-bearing mice. **(i-j)** Comparison of CD4^+^ T cells in tumor tissue (i) and spleen tissue (j) on the 12th day after the start of treatment. Data are presented as mean ± standard deviation (*n* = 3), *p < 0.05, **p < 0.01, ***p < 0.001

Cells undergoing ICD can stimulate DCs maturation by exposing CRT to the cell surface and binding to CD91 on the DCs [35]. In this study, we detected the proportion of mature DCs in tumor tissues using flow cytometry and the results are shown in Fig. 10a, the proportion of mature DCs in tumor tissues was 0.86% (saline), 0.92% (Cur), 1.04% (PPH@C), 1.06% (Cur + Rg3) and 1.34% (PPH@CR), respectively, with the PPH@CR group having the highest proportion of mature DCs. And we also measured the proportion of mature DCs in the spleen of mice (Fig. 10b), and the proportion of mature DCs in the spleen of mice was 0.47% (saline), 0.92% (Cur), 1.41% (PPH@C), 1.61% (Cur + Rg3) and 1.92% (PPH@CR), respectively, which were consistent with the trend of the proportion of mature DCs cells in tumor tissues, and the proportion of mature DCs cells in spleen tissues in the PPH@CR group was still the highest. The above results indicated that PPH@CR could increase the proportion of mature DCs in mice, which is beneficial for activating anti-tumor immune responses.

### PPH@C and PPH@CR promote effector T cell proliferation in vivo

Tumor cells undergoing ICD can be used as vaccines or as immune adjuvants to attract and promote DCs maturation, which in turn acts as powerful antigen-presenting cells (APCs) to activate adaptive immune responses, particularly CD8 killer T cells and CD4 helper T cells [4]. In this study, we detected the proportion of infiltrative CD8^+^ T cells in tumor tissues using flow cytometry and the results are shown in Fig. 10c, the number of CD8^+^ T cells in the tumor tissues of tumor-bearing mice accounted for 3.18% of the total number of cells in the saline group, and this value increased to 3.95%, 5.2%, 6.85% and 15.36% after Cur, PPH@C, Cur + Rg3 and PPH@CR treatment, respectively, indicating nanomicelle PPH@CR could effectively increase the proportion of tumor-infiltrating CD8^+^ T cells. In addition, the proportions of CD8^+^ T cells in the spleen of mice was 6.52% (normal saline), 7.2% (Cur), 8.34% (PPH@C), 8.29% (Cur + Rg3), and 11.94% (PPH@CR), respectively. In summary, PPH@CR could significantly increase the proportion of CD8^+^ T cells in tumor tissues and spleen tissues, indicating that it can effectively activate anti-tumor immune responses and improve tumor killing ability.

CD4^+^ T cells are the foundation of protective immunity which not only express molecules related to cell lysis such as granzyme (GZM) and perforin (PRF1) but also have direct cytotoxic effects [36]. Therefore, we detected CD4^+^ T cells in each group of tumor tissues using flow cytometry and the results are shown in Fig. 10c, the number of CD4^+^ T cells in the tumor tissues of tumor-bearing mice accounted for only 0.36% of the total number of cells in the saline group, while the number of CD4^+^ T cells in the other groups increased significantly, among them, CD4^+^ T cells accounted for the largest proportion in the PPH@CR group (2.55%), followed by the Cur + Rg3 group (1.1%), the PPH@C group (0.74%) and the Cur group (0.64%). And the proportion of CD4^+^ T cells in the spleen tissues of mice were further detected and the results are shown in Fig. 10d, the proportions of CD4^+^ T cells in the spleen tissues of each group was 19.3% (saline group), 21.1% (Cur group), 25.1% (PPH@C group), 26.58% (Cur + Rg3 group) and 29.06% (PPH@CR group). Consistent with the trend of the proportion of CD4^+^ T cells in tumor tissues, the proportion of CD4^+^ T cells in spleen tissues was the highest in the double-drug micelle PPH@CR group, which was significantly better than that in other groups. The above results showed that the nanomicelles PPH@C and PPH@CR could increase the proportion of effector T cells in the tumor microenvironment compared with free drugs, so that the tumor immune microenvironment changes from an immune-tolerant state to an immunoactivated state, and the relatively “cold” tumor TNBC becomes a “hot” tumor infiltrated by T cells, which in turn helps to activate the anti-tumor immune response to kill tumor cells and inhibit tumor growth.

### In vivo safety

**Fig. 11:**
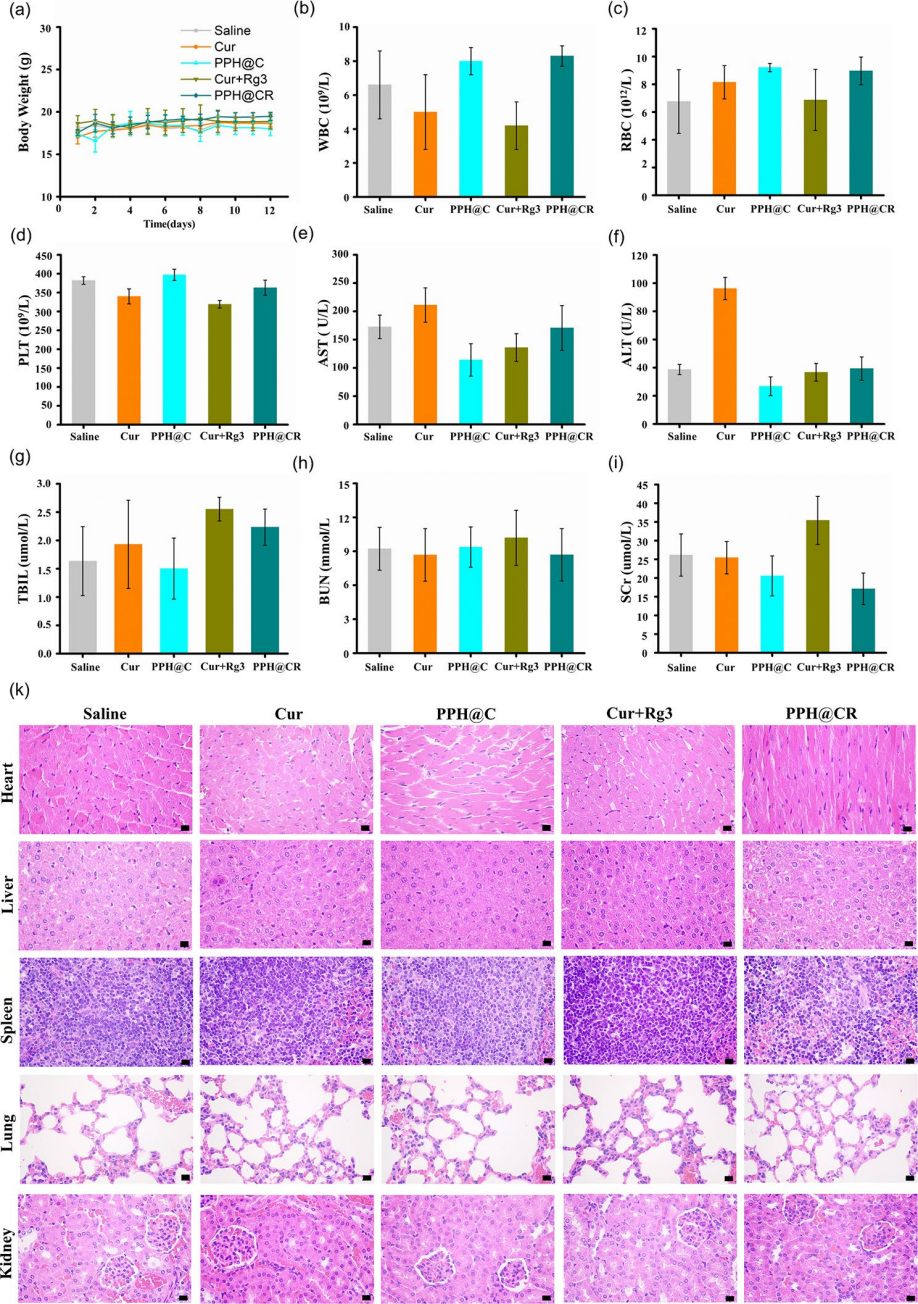
Biocompatibility of free drug and nanomicelles for BALB/c mice. **(a)** The body weight curves of tumor-bearing mice in each group (saline, Cur, PPH@C, Cur + Rg3 and PPH@CR group) after drug treatment. (b-d) Quantification of routine blood (including WBC, RBC, PLT) of tumor-bearing mice in each group after drug treatment. (e-i) Quantification of liver and kidney function indicators (AST, ALT, T-BIL, BUN, SCr) in tumor-bearing mice in each group after drug treatment. **(k)** The H&E staining images of organ tissue sections of heart, liver, spleen, lung, and kidney of mice in each group after treatment (Scale bar, 20 μm). All data are presented as mean ± standard deviation (*n* = 3)

We monitored mouse body weight in order to assess the safety of PPH@CR in vivo and found that the average body weight of mice in all groups remained stable during and after treatment (Fig. 11a). The hematologic toxicity of the mice was further evaluated, and the results are shown in Fig. 11(b-i), and the blood routine (Fig. 11b-d) and liver and kidney function indexes (Fig. 11e-i) of the tumor-bearing mice in each group were within the normal range compared with the control group. In addition, we also evaluated the toxicity of the drug to the major organs of the mice in each group, and the results are shown in Fig. 11k, and no significant damage and lesions were observed in the major organs of the mice collected in each group. All of the above results indicated that our synthetic nanomicellars PPH@C and PPH@CR have good biosafety and can be used for the treatment of cancer.

## Discussion

Breast cancer is the most common malignant tumor in women, and the treatment of metastatic TNBC is still a comprehensive treatment based on chemotherapy due to the lack of effective therapeutic targets for TNBC. And it is particularly important to find anti-tumor treatment drugs with high selectivity and few side effects due to the shortcomings of traditional chemotherapy drugs, such as low specificity, high drug resistance rate and systemic toxicity. In addition to traditional chemotherapy drugs, there are also a variety of anti-tumor active substances in nature, such as Cur and glycoside Rg3 extracted from natural products, which can exert anti-tumor effects through a variety of mechanisms, such as inducing ICD in tumor cells, reducing tumor PD-L1 protein expression and reducing cancer stem cells. However, these two drugs also have the characteristics of poor water solubility, low bioavailability, and weak anti-tumor effects, so finding strategies to overcome these shortcomings is the key to give full play to the anti-tumor effects of the two drugs.

The nanodrug delivery system can achieve efficient drug delivery by loading multiple drugs and has the effect of enhancing anti-tumor efficacy. Among them, N-O zwitterionic polymers are ideal drug delivery vehicles, which have been shown to have good biocompatibility and transphagocytosis. The vinyl ether bonds formed by the click reaction of acylalkyne monomers with phenolic drugs can be broken down at low pH, which is a very effective method for pH-responsive drug delivery. Based on this, we designed and synthesized the N-O zwitterionic polymer OPDEA-PGED, which linked Cur to the N-O zwitterionic polymer by vinyl ether bonding and encapsulated ginsenoside Rg3 to obtain hyperbranched zwitterionic drug-loaded micelles OPDEA-PGED-5HA@Cur@Rg3 (PPH@CR), among them, Cur is not only an anti-tumor drug but also a cross-linking agent of drug-loaded micelles. It was further confirmed that the PPH@CR was a homogeneous spherical structure with ideal size and zeta potential using TEM, DLS and zeta potentiometer. Moreover, it has good stability and has the characteristics of responding to tumor low-pH to release drugs, which is conducive to the release of loaded drugs by drug-loaded micelles PPH@CR in the low-pH tumor microenvironment thereby playing a role in targeting tumors.

In addition, in vitro cell experiments and in vivo experiments in tumor-bearing mice have shown that PPH@CR can promote the maturation of DCs and the proliferation of CD4^+^ T cells and CD8^+^ T cells by inducing ICD in tumor cells, so that the tumor microenvironment of relatively “cold” TNBCs can be transformed into a “hot” tumor microenvironment infiltrated by immune cells. In addition, PPH@CR can also reduce the expression of PD-L1 protein in tumor tissues, which is conducive to alleviating the immune escape caused by PD-L1/PD-1 signaling, and promoting T cell activation and T cell killing in tumor tissues. At the same time, PPH@CR also has the effect of reducing cancer stem cells, which is conducive to reducing tumor recurrence, tumor metastasis and treatment resistance. In conclusion, drug-loaded micelles PPH@CR loaded with Cur and Rg3 can exert anti-tumor effects through a variety of mechanisms, not only that, it has stronger anti-tumor effects than free drugs, which is a promising cancer treatment strategy.

## Conclusion

Our synthetic drug-loaded micelle PPH@CR can effectively deliver the poorly water-soluble ICD inducers Cur and Rg3 to tumor tissues, activate autologous anti-tumor immune responses, and improve anti-tumor efficacy, which is a promising cancer treatment strategy.

### Electronic supplementary material

Below is the link to the electronic supplementary material.

Supplementary Material 1

## Data Availability

No datasets were generated or analysed during the current study.

## Abbreviations

TNBC: Triple negative breast cancer
PD-L1: Programmed death ligand 1
Cur: Curcumin
ICD: Immunogenic cell death
DCs: Dendritic cells
DAMPs: Damage-associated molecular patterns
PD-1: Programmed cell death 1
ATRP: Atom transfer radical polymerization
HMGB1: High-Mobility Group Box 1
ATP: Adenosine Triphosphate
PPH: OPDA-PGED-5HA
PPH@C: OPDEA-PGED-5HA@Cur
PPH@CR: OPDEA-PGED-5HA@Cur@Rg3
DLS: Dynamic light scattering instrument
UV: Ultraviolet spectrophotometer
TEM: Transmission electron microscopy
CRT: Calreticulin
