# Supplementary material for: A pH-triggered N-oxide polyzwitterionic nano-drug loaded system for the anti-tumor immunity activation research
Source: J Nanobiotechnology. 2024 Jul 16;22:420. doi: 10.1186/s12951-024-02677-0 (PMC11253471; doi:10.1186/s12951-024-02677-0)
Supplement: Supplementary file 1 — Supplementary Material 1 [file 12951_2024_2677_MOESM1_ESM.docx]

Supporting Information

# A pH-triggered N-oxide polyzwitterionic nano-drug loaded system for the anti-tumor immunity activation research

Yan Zhao^1^, Yuansong Bai^1✉^, Mei Li^1^, Xin Nie^2^, Hao Meng^3^, Shimizu Shosei^4,5^, Linlin Liu^3^, Qingbiao Yang^6^, Meili Shen^3✉^, Yapeng Li^6✉^

^1^Department of Oncology and Hematology, China-Japan Union Hospital of Jilin University, Changchun, Jilin, 130033, China.

^2^Stroke center, Jilin Electric Power Hospital, Changchun, Jilin, 130022, China.

^3^Department of Radiation Oncology, China-Japan Union Hospital of Jilin University, Changchun, Jilin, 130033, China.

^4^Pediatric Radiation Therapy Center/Pediatric Proton Beam Therapy Center, University of Tsukuba Hospital, Tsukuba,3050005, Japan.

^5^Hebei Yizhou Cancer Hospital. Zhuozhou, Hebei, 072750, China.

^6^Key Laboratory of Special Engineering Plastics Ministry of Education, College of Chemistry, Jilin University, Changchun, Jilin, 130012, China.

Corresponding authors: Yuansong Bai, [baiys@jlu.edu.cn](mailto:baiys@jlu.edu.cn); Address: Department of Oncology and Hematology, China-Japan Friendship Hospital, Jilin University, Changchun, Jilin, 130033, China. Meili Shen, shenmeili@jlu.edu.cn. Department of Radiation Oncology, China-Japan Union Hospital of Jilin University, Changchun, Jilin, 130033, China. Yapeng Li, [liyapeng@jlu.edu.cn](mailto:liyapeng@jlu.edu.cn). Key Laboratory of Special Engineering Plastics Ministry of Education, College of Chemistry, Jilin University, Changchun, Jilin, 130012, China. Tel./Fax: +86 431 85168238.

## Materials and methods

### Materials

2-(Diethylamino)ethyl methacrylate (DEA), glycidyl methacrylate (GMA), 2,2'-Bipyridyl (bpy), cuprous chloride (CuCl), 4-Dimethylaminopyridine (DMAP), HBTU, N,N-Diisopropylethylamine (DIPEA), Cur, ginsenoside Rg3, genistein, 5-(N,N-Hexamethylene)-amiloride, ethyl α-bromoisobutyrate, 5-Hexynoic acid (5-HA) were purchased from Aladdin Chemistry Co, Ltd. (Shanghai, China). HOBt and wortmannin were ordered from Macklin Biochemical Technology Co, Ltd. (Shanghai, China). 30% hydrogen peroxide and chlorpromazine were ordered from Kaiyuan, China and J&K Chemical. (Beijing, China), respectively. Mouse High-Mobility Group Box 1 (HMGB1) ELISA Kit, Mouse Adenosine Triphosphate (ATP) ELISA Kit, Calreticulin Polyclonal Antibody, HMGB1 Polyclonal Antibody, CD44 Antibody, and PD-L1 Antibody were sourced from Mitaka Biotechnology Co, Ltd. (Wuhan, China). PE Anti-Mouse CD86 Antibody, APC Anti-Mouse CD11C Antibody, PerCP Anti-Mouse CD4 Antibody, FITC Anti-Mouse CD8a Antibody and PE anti-mouse CD274/PD-L1 antibody were purchased from Elabscience. (Wuhan, China).

### Synthesis of the PDEA-PGMA

PDEA-PGMA was prepared by atom ATRP [1]. Briefly, 10 mL of DMF was added to a 50 mL branched round-bottom flask containing 0.064 g CuCl and 0.064 g bpy. Then 12 mL DEA, 6mL GMA, and 270 μL 2-bromoisobutyrate ethyl ester were added to the above solution under degassing conditions. And added dichloromethane for quenching reaction after the reaction was carried out at 55 °C in argon atmosphere for 5 h. The catalyst was removed from the alumina column and the solvent was removed by vacuum distillation, then the crude product was recrystallized with cold n-hexane and vacuum-dried to obtain 22 g of PDEA-PGMA. The synthetic route is shown in Fig. S1.

**Synthesis of the PDEA-PGED**

PDEA-PGED was synthesized based on previous literature with some modifications [1]. Firstly, the obtained PDEA-PGMA (16 g) was dissolved in 100 mL of DMSO and added excess EDA and the reaction was carried out at 80 °C for 4 h in a nitrogen atmosphere. Then the reaction was dialysed in deionized water (MWCO: 1000 Da) for 48 h to remove excess EDA. Finally, the final product PDEA-PGED (12.5 g, pale yellow powder) was obtained by lyophilization. The synthetic route is shown in Fig. S1.

**Synthesis of the OPDEA-PGED**

PDEA-PGED was oxidized with reference to previous literature [2]. Firstly, PDEA-PGED (9 g) was dissolved in 100 mL of 30% H_2_O_2_ with stirring for 4 h at room temperature, then dialysed with deionized water (MWCO: 3500 Da) to remove excess H_2_O_2_. Finally, the polymer OPDEA-PGED (8.2 g, pale yellow powder) was obtained by lyophilization. The synthetic route is shown in Fig. S1.

**Synthesis of the OPDEA-PGED-5HA**

OPDEA-PGED-5HA was synthesized based on previous literature with some modifications [3]. Firstly, 5-hexynic acid (10 g, 89 mmol), HBTU (15 g, 40 mmol), DIPEA (22 g, 172 mmol), and HOBt (6 g, 44.0 mmol) were dissolved in anhydrous DMF (20 mL) and stirred 30 min at 0 °C in a nitrogen atmosphere. Followed by the addition of OPDEA-PGED (6 g) and the reaction continued for 12 h at 25 °C. Then dialyzed with deionized water (MWCO: 3500 Da) to remove the catalyst and excess 5-HA. Finally, the polymer OPDEA-PGED-5HA (8.73 g, brown powder) was obtained by lyophilization. The synthetic route is shown in Fig. S1.


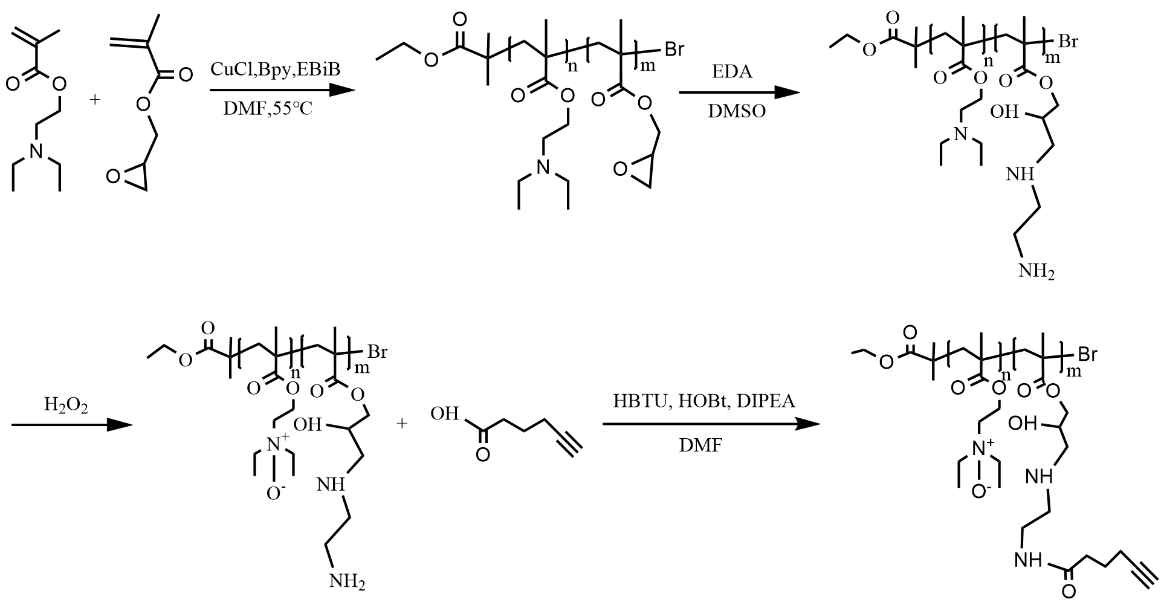


**Fig. S1** Synthesis route of OPDEA-PGED-5HA

**Preparation of empty micelles PPH and drug-loaded micelles**

Blank micelles OPDA-PGED-5HA (PPH) were prepared according to the solvent exchange method. Firstly, 10.0 mg of OPDA-PGED-5HA was dissolved in 2.0 mL of tetrahydrofuran (THF) and dropwise added to 8.0 mL of distilled water at 25 °C. Then the resulting mixture was dialysed with deionized water (MWCO: 1000 Da) for 24 h to obtain empty micelles PPH.

Preparation of Cur-loaded micelles OPDEA-PGED-5HA@Cur (PPH@C) [4]. 10.0 mg of OPDA-PGED-5HA, 3.7 mg of Cur and 0.5 mg of DMAP were dissolved in 2.0 mL THF as shown in Fig. S2 and stired for 24 h at 37 °C. The mixed solution was added dropwise to 8.0 mL of stirred distilled water at 25 °C and dialyzed with deionized water (MWCO: 1000 Da) to remove THF, catalyst, and finally centrifugation to remove unbound curcumin to obtain drug-loaded micelles PPH@C.

Micellars OPDEA-PGED-5HA@Cur@Rg3 (PPH@CR) loaded with both Cur and ginsenoside Rg3 were prepared by a similar method. 10.0 mg of OPDA-PGED-5HA, 3.7 mg of Cur, 3.0 mg of Rg3, and 0.5 mg of DMAP were dissolved in 2.0 mL THF as shown in Fig. S2 and stirred for 24 h at 37 °C. Then the mixed solution was added dropwise to 8.0 mL of distilled water and dialyzed with deionized water (MWCO: 1000 Da) to remove THF, catalyst, unbound Cur and Rg3, and finally centrifugation to remove unbound Cur and Rg3 to obtain drug-loaded micelles PPH@CR.


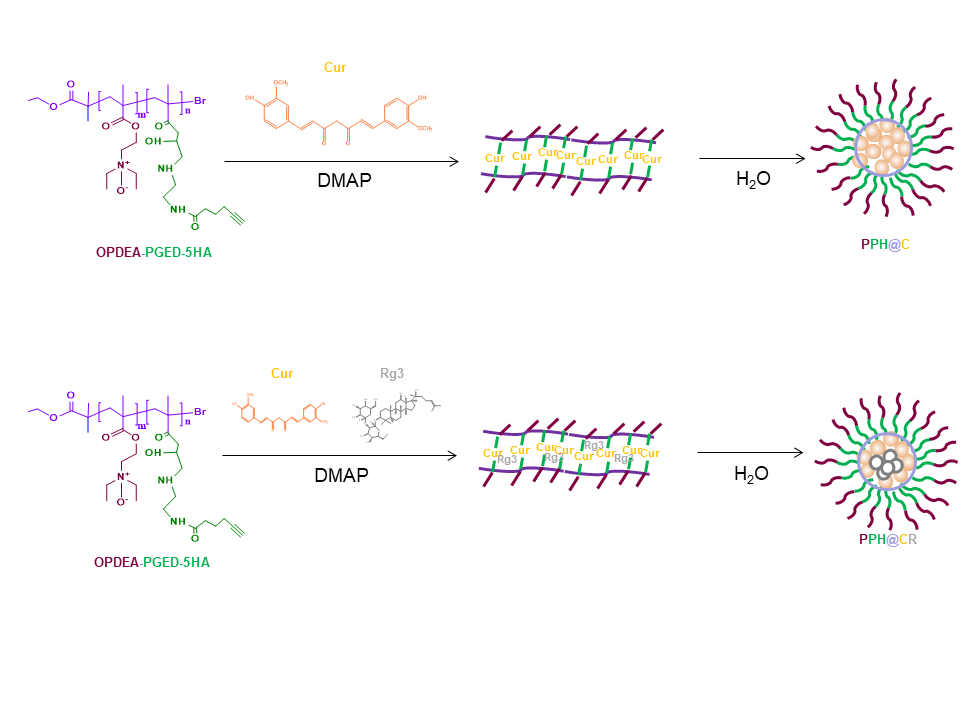


**Fig. S2** Synthetic routes of drug-loaded micelles PPH@C and PPH@CR

**Characterization of material**

The chemical structures of PDEA-PGMA, PDEA-PGED, OPDEA-PGED, and OPDEA-PGED-5HA were characterized using ^1^H-NMR spectroscopy (300 MHz, CDCl_3_) and Fourier transform infrared spectroscopy (FT-IR), respectively.

**Characterization of micelles**

The particle size and zeta potential of empty micelles PPH as well as drug-loaded micelles PPH@C and PPH@CR in distilled water were determined using dynamic light scattering instrument (DLS) at 25 °C. The morphology and size of micelles were investigated by high-resolution transmission electron microscopy (TEM, JEOL-JEM-2100F). Briefly, placed the copper mesh on a stable plane and aspirated 10 μL of PPH, PPH@C, and PPH@CR with a syringe and dropped on a 300-mesh copper mesh, respectively, to form water droplets and let them dry naturally before taking TEM photos [6]. In order to test the stability of PPH, PPH@C, and PPH@CR, the synthesized empty micelles and drug-loaded micelles were exposed to saline and stored at 4 °C, and particle size and zeta potentials were detected on days 1, 5, 10, 20, and 30, respectively.

### Drug loading of PPH@C and PPH@CR

The ultraviolet peaks of Cur and Rg3 in micelles were detected by ultraviolet spectrophotometer (UV) at 25 °C after placing PPH@C and PPH@CR in deionized water for 30 minutes at pH 5.5 (The UV absorption peak of curcumin was 425 nm and the UV absorption of Rg3 was 203 nm) [7]. and the drug loading was calculated according to the established standard curve. All data were presented as averages and the trial was repeated three times. Drug loading (DL%) and encapsulation rate (EE%) were calculated using equations (1) and (2), respectively [8]：

$$\mathrm{DL}\left( \% \right)=\frac{The amount of drug in the nanoparticle}{Total mass of nanoparticles} \cdots\cdots\cdots\cdots\cdots\cdots(1)$$

$$\mathrm{EE}\left( \% \right)=\frac{The amount of drug in the nanoparticle}{Total dosage} \cdots\cdots\cdots\cdots\cdots\cdots(2)$$

### Drug releasing of PPH@CR

The PPH@CR can respond to low pH due to the presence of vinyl ether bonds. So we prepared saline dialysate of different pH values (7.4, 6.5, 5.5) containing 0.1% (v/v) tween 80 to simulate the normal physiological environment, tumor microenvironment and lysosomal internal environment [9, 10]. Then immersed dialysis tubing (MWCO: 3500 Da) containing equal amounts of PPH@CR solution to 50 mL of dialysate of different pH values (pH = 7.4 or pH = 5.5) [11] and slowly stired them in a dark environment at 37 ℃. Then removed 3 mL of the solution on the outside of the dialysis bag at different time points, and added the same volume of fresh dialysate corresponding to the pH value to keep the volume of the solution outside the dialysis bag constant. The emission of Cur and Rg3 were determined with UV and calculated from the established standard curve (Fig. S1). All data were presented as averages and the trial was repeated three times. The cumulative release (C_A_) was calculated according to the following equation [11].

$$C_{A}=\frac{M_{t}}{M_{\mathrm{total}}}\times100\%$$

In this equation, M_t_ represents the amount of drug released from the nanoparticles at different times, and M_total_ represents the total amount of drug in the nanoparticles.

We also observed the morphology, size, and zeta potential of two drug-loaded nanoparticles at different pH environments. Briefly, PPH@C and PPH@CR were placed in saline at pH 7.4 and pH 5.5, respectively. And the morphological changes of micelles were observed using TEM, and the changes in particle size and zeta potential of PPH@C and PPH@CR at different pH were measured using DLS and zeta potentiometers.

**Cells and animals**

The triple-negative breast cancer cell line 4T1 was donated by Changchun Institute of Applied Chemistry, Chinese Academy of Sciences, and cultured in RPMI 1640 medium containing 10% fetal bovine serum and 1% penicillin-streptomycin in a cell culture incubator containing 5% CO_2_ at 37 °C [12]. Female BALB/c mice (6-8 weeks old) were purchased from Beijing Weitong Lihua Company, and were raised at 20 °C without specific pathogens (SPF) and allowed to acclimatize in a rearing facility for at least 7 days, and had free access to water and food before the experiment. All animal experimental protocols were approved by the Animal Ethics and Experiment Committee of Jilin University (approval number：2024 extension No. 3).

### Cytotoxicity of PPH@C and PPH@CR

The effects on tumor cell proliferation of empty micelles PPH and drug-loaded micelles PPH@C and PPH@CR were evaluated by CCK-8 kit. Briefly, the experiment was divided into 5 groups (PPH, Cur, PPH@C, Cur + Rg3 and PPH@CR group), and 4T1 cells (3 × 10^3^ cells/well) were inoculated and cultured in 96 well plate for 24 h, then incubated with different concentrations of PPH (0 μg/mL, 25 μg/mL, 50 μg/mL, 75 μg/mL, 100 μg/mL, 200 μg/mL), Cur, PPH@C, Cur + Rg3, and PPH@CR (the last 4 groups contained the same amount of Cur or Rg3, where Cur concentrations were 0 μg/mL, 5 μg/mL, 10 μg/mL, 20 μg/mL, 50 μg/mL and 100 μg/ mL, the concentrations of Rg3 were calculated according to the drug load of Rg3 in the PPH@CR, respectively: 0 μg/mL, 0.7 μg/mL, 1.4 μg/mL, 2.8 μg/mL, 5.6 μg/mL, 7.1 μg/mL, 14.3 μg/mL). Subsequently, 10 μL of CCK-8 reagent was added to each well and incubated for 1 h. Then the absorbance at 450 nm was determined using microplate reader and IC_50_ values were calculated using CompuSyn.

The effect of each group of drugs on cell viability was evaluated with the following cell survival formula [13]:

$$Cell viability(\%)=\frac{\mathrm{OD}_{s}-\mathrm{OD}_{b}}{\mathrm{OD}_{c}-\mathrm{OD}_{b}}\times100\%$$

Among them, *ODs* and *OD_C_*are the absorbance values of the test sample and the untreated sample, respectively. *OD_b_* is the absorbance of RPMI1640 culture medium without cells.

***In vitro* cell uptake**

The entry of drugs into cells is crucial for achieving therapeutic effects. We observed the uptake of Cur, PPH@C, and PPH@CR by 4T1 cells using the intrinsic fluorescence spectrum of Cur, and the amount of Cur in the cells was proportional to the fluorescence intensity. Briefly, 4T1 cells were inoculated in 6-well plates (5.0 × 10^5^ cells/well) and cultured with RPMI 1640 at 37 ℃, then Cur, PPH@C and PPH@CR were added after the cells were attached (the concentration of Cur in each group was 50 μg/mL) and co-cultured for 0.5h, 2h and 6h. Then washed off unbound drugs and nanoparticles with PBS and fixed cells with 4% paraformaldehyde (PFA) for 30 min. Nuclei were stained with DAPI for 5 min in a dark environment, then the dye was washed off and the cells were imaged by fluorescence microscopy under excitation at 425 nm [14, 15], and the fluorescence intensity of Cur was quantified using ImageJ (Bethesda, MD).

**Mechanism of uptake of PPH@C and PPH@CR**

We further investigated the uptake mechanism of Cur, drug-loaded micelles PPH@C and PPH@CR. 4T1 cells were seeded in 6-well plates (5.0 × 10^5^ cells/well) and incubated for 24 h, and pretreated with the endocytic inhibitors chlorpromazine (8.5 μg/mL), genistein (56.75 μg/mL), 5-(N,N-Hexamethylene)-amiloride (133 μg/mL), and wortmannin penicillin (5 μmol) for 1 h [2, 16]，followed by Cur, PPH@C, or PPH@CR (the concentration of Cur was 50 μg/mL) for 2 h. Finally, the uptake of drugs by 4T1 cells in each group was analyzed by flow cytometry.

**Exposure of CRT in 4T1 cells**

In this experiment, we assessed the ICD condition induced by PPH@C and PPH@CR in tumor cells by detecting exposure to calreticulin (CRT) in tumor cells. Briefly, 4T1 cells (5.0 × 10^4^ cells/well) were seeded in pre-treated 24-well plates containing coverslips for 24 h, and drugs (the concentrations of Cur and Rg3 were 50 μg/mL and 7.1 μg/mL, respectively) were added to each group (PBS, Cur, PPH@C, Cur + Rg3, and PPH@CR group) to continue cultured 24 h. Then CRT primary antibodies were added and incubated overnight at 4 °C, followed by fluorescence-coupled secondary antibodies for 1 h, and finally DAPI was added for 10 min, glycerol mounted, and CRT expression was observed by fluorescence microscopy.

***In vitro* detection of ATP and HMGB1**

ATP and HMGB-1 in 4T1 cells induced by PPH@C and PPH@CR. The experiment was also divided into 5 groups (PBS, Cur, PPH@C, Cur + Rg3 and PPH@CR group). 4T1 cells were seeded in 6-well plates at a density of 5 × 10^5^ cells/well for 24 h, administered (Cur and Rg3 were administered at concentrations of 50 μg/mL and 7.1 μg/mL, respectively) and continue incubated for 24 h. Then the levels of ATP and HMGB1 released by cells in each group were measured using mouse ATP ELISA kit and HMGB-1 ELISA kit, respectively.

### Effects of PPH@C and PPH@CR on DCs maturation and PD-L1 in 4T1 cells

4T1 cells were seeded on the basolateral side of the transwell plate (5.0 × 10^5^ cells/well) and incubated for 24 h, and the experiment was divided into 5 groups, including PBS, Cur, PPH@C, Cur + Rg3 and PPH@CR groups, and the drugs in each group were added (the administration concentration of Cur was 50 μg/mL, and the administration concentration of Rg3 was 7.1 μg/ mL), then DCs were seeded at the apex after 24 h, and continue to culture for 48 hours before collecting DCs, and APC-labeled anti-mouse CD11c antibody (0.5 μg/10^6^ cells, Elabscience), PE-labeled anti-mouse CD86 antibody (0.5 μg/10^6^ cells, Elabscience) were added, and the expression of CD11c and CD86 on DCs were analyzed by flow cytometry (FCM, China, Lamina), and the obtained data were analyzed using V3.0.1.4609 software (Strata Biotech, China).

4T1 cells were seeded in 6-well plates (5.0 × 10^5^ cells/well) and incubated for 24 h, and the experiment was also divided into 5 groups,, including PBS, Cur, PPH@C, Cur + Rg3 and PPH@CR groups, and the drugs in each group were added (the administration concentration of Cur was 50 μg/mL, and the administration concentration of Rg3 was 7.1 μg/mL) and continue to culture for 48 hours, then the cells were washed with PBS for three times. PE Anti-Mouse CD274/PD-L1 Antibody (0.5 μg/10^6^ cells, Elabscience) was added, finally PD-L1^+^ 4T1 cells were analyzed by flow cytometry, and the obtained data were analyzed using V3.0.1.4609 software.

**Scratch experiment**

Cancer cells have the ability to migrate and invade. In this study, we evaluated the migration effects of PPH@C and PPH@CR on tumor cells using scratch assays. The experiments were divided into 5 groups: PBS, Cur, PPH@C, Cur + Rg3 and PPH@CR group (Cur and Rg3 were administered at concentrations of 50 μg/mL and 7.1 μg/mL, respectively). First, 4T1 cells were seeded in 6-well plates (5 × 10^5^/well) and incubated for 24 h at 37 °C, 5% CO_2_, then scratched in cell monolayers using a 200 μLpipette tip and images of the scratches were taken and measured with a microscope at 0 h, and each group of drugs was added and incubated for 24 h and 48 h, respectively, and the cell-free regions after incubation were imaged and measured using microscope [17].

***In vivo* antitumor efficacy of PPH@C and PPH@CR**

Animal models of tumor-bearing mice were established. First, 4T1 cells (1 × 10^6^) were suspended in 100 μL of PBS and injected subcutaneously into the right axillary region of healthy BALB/c mice to develop solid tumors. And the tumor-bearing mice were randomly divided into 5 groups (n = 5) when the tumor volume reached about 100 mm^3^, which were saline, Cur, PPH@C, Cur + Rg3, PPH@CR, respectively. Each group was injected through the tail vein for a total of 6 times, which were administered on days 1, 3, 5, 7, 9 and 11, and the administered dose of Cur was 20 mg/kg and the administered dose of Rg3 was 2.8 mg/kg (calculated based on the drug load of Rg3 in the PPH@CR). The length and width of tumors were measured daily using vernier calipers while monitoring the body weight of the mice. And the mice were sacrificed on the 12th day after initiation of treatment, then the tumor tissues of the mice in each group were collected and fixed with 4% formaldehyde, then hematoxylin-eosin staining (H&E staining) was performed and the cell morphology of the tumor tissues were observed using light microscopy. The tumor volume and the tumor inhibition rate (TIR) in mice were calculated according to the following formula, respectively [18]：

$$V=L\times W^{2}\times0.5$$

In this formula, V represents the tumor volume, L represents the tumor length, and W represents the tumor width.

$$TIR=\frac{W_{C}-W_{T}}{W_{C}}\times100\%$$

In this formula, TIR represents the tumor inhibition rate, *W_C_* represents the average tumor weight of the control group, and W_T_ represents the average tumor weight of the treatment group.

### Detection of Ki67, CD44 and PD-L1 in tumor tissues

The tumor tissues of mice treated with each group of drugs (saline, Cur, PPH@C, Cur + Rg3 and PPH@CR) were collected on the 12th day after treatment，and fixed them with 4% formaldehyde, embedded in paraffin and made into sections. Then the diluted Ki67 (1:600), CD44 (1:800) and PD-L1 (1:1000) antibodies were added dropwise to the sections and incubated overnight at 4 °C. Subsequently, secondary antibodies were added dropwise on the sections and incubated for 50 min at room temperature in the dark, and DAB chromogenic agent was added dropwise, the sections were counterstained with hematoxylin after the color was completed, then dehydrated and sealed. Finally, the expressions of Ki67, CD44 and PD-L1 in tumor tissues of tumor-bearing mice were analyzed by microscopy.

### Detection of CRT and HMGB1 in tumor tissues

We further examined the expression of CRT and HMGB1 in tumor tissues using immunofluorescence staining to evaluate the induction of ICD in tumor cells by PPH@C and PPH@CR *in vivo* [19]. Briefly, tumor tissues from mice in each group were collected on day 12 after treatment and fixed with 4% paraformaldehyde. The anti-CRT primary antibody (diluted 1:200) or HMGB1-antibody (diluted 1:200) was added dropwise to the sections and placed in a 4 °C freezer overnight. Then the secondary antibody was added dropwise and the nuclei was counterstained with DAPI for 50 minutes. Finally, observed and took images using fluorescence microscopy.

**Detection of dendritic cells *in vivo***

Cells undergoing ICD can promote the transformation of DCs to mature phenotype by releasing DAMPs, and mature DCs are the main antigen-presenting cells in the human body and have the characteristics of MHCII and CD86 [20]. We detected mature DCs in tumor tissue by flow cytometry in this study, briefly, the collected tumor tissues from each group of mice were placed in culture dishes containing medium and cut into small pieces, then digested using medium containing type IV collagenase (100 U/mL) to obtain a single-cell suspension of tumor tissue, and APC-labeled anti-mouse CD11c antibody (0.5 μg/10^6^ cells, Elabscience), PE-labeled anti-mouse CD86 antibody (0.5 μg/10^6^ cells, Elabscience) were added to the cell suspension and incubated on ice for 40 min in the dark. Finally, the expression of CD11c and CD86 on DCs were analyzed by flow cytometry.

We further examined mature DCs in mouse spleen tissue. First, the collected mice spleens were minced and sieved, and the cells were resuspended using pre-chilled erythrocyte lysate and incubated on ice for 5 min. Centrifuged the cells again and discarded the supernatant and added APC-labeled anti-mouse CD11c antibody (0.5 μg/10^6^ cells, Elabscience), PE-labeled anti-mouse CD86 antibody (0.5 μg/10^6^ cells, Elabscience), and incubated on ice for 40 min in the dark. Finally, the expression of CD11c and CD86 on DCs were analyzed by flow cytometry and the obtained data were analyzed using V3.0.1.4609 software (Stratus Biotech, China).

**Detection of CD8^+^ T cells and CD4^+^ T cells *in vivo***

ICD induces DCs maturation and further activates adaptive immune responses, including the proliferation of cytotoxic T lymphocytes (CD8^+^ T lymphocytes) and helper T lymphocytes (CD4^+^ T lymphocytes) [21]. In this study, we examined the ratio of CD8^+^ T cells and CD4^+^ T cells in tumor tissues as well as spleen tissues using flow cytometry [22]. First, the tumor tissues and spleens of mice were isolated and prepared into cell suspension (Look at the " Detection of dendritic cells *in vivo*" section) on the 12th day after the start of treatment. Then PITC-labeled anti-CD8 antibody (0.2 μg/10^6^ cells, Elabscience) and PerCP-labeled anti-CD4 antibody (0.25 μg/10^6^ cells, Elabscience) were added to the prepared single-cell suspension to distinguish between CD8^+^ T lymphocytes and CD4^+^ T lymphocytes and incubated on ice for 40 min in the dark. Finally, the test was performed by flow cytometry and the obtained data was analyzed.

### *In vivo* safety

Venous blood was collected from mice for biochemical and complete blood count testing before the end of treatment. And the main organs of mice (heart, liver, spleen, lung and kidney) were collected at the end of treatment, and fixed with 4% paraformaldehyde and stained with H&E to evaluate the histomorphological changes of the main organs of mice after treatment.

### Statistical analysis

Statistical analysis was performed using GraphPad Prism software, and all data were expressed as mean ± standard deviation. The t-test was used to analyze the statistical differences between the groups, and *p<0.05 was considered statistical difference, **p<0.01 was considered significant difference, and ***p<0.001 was considered extremely significant difference.


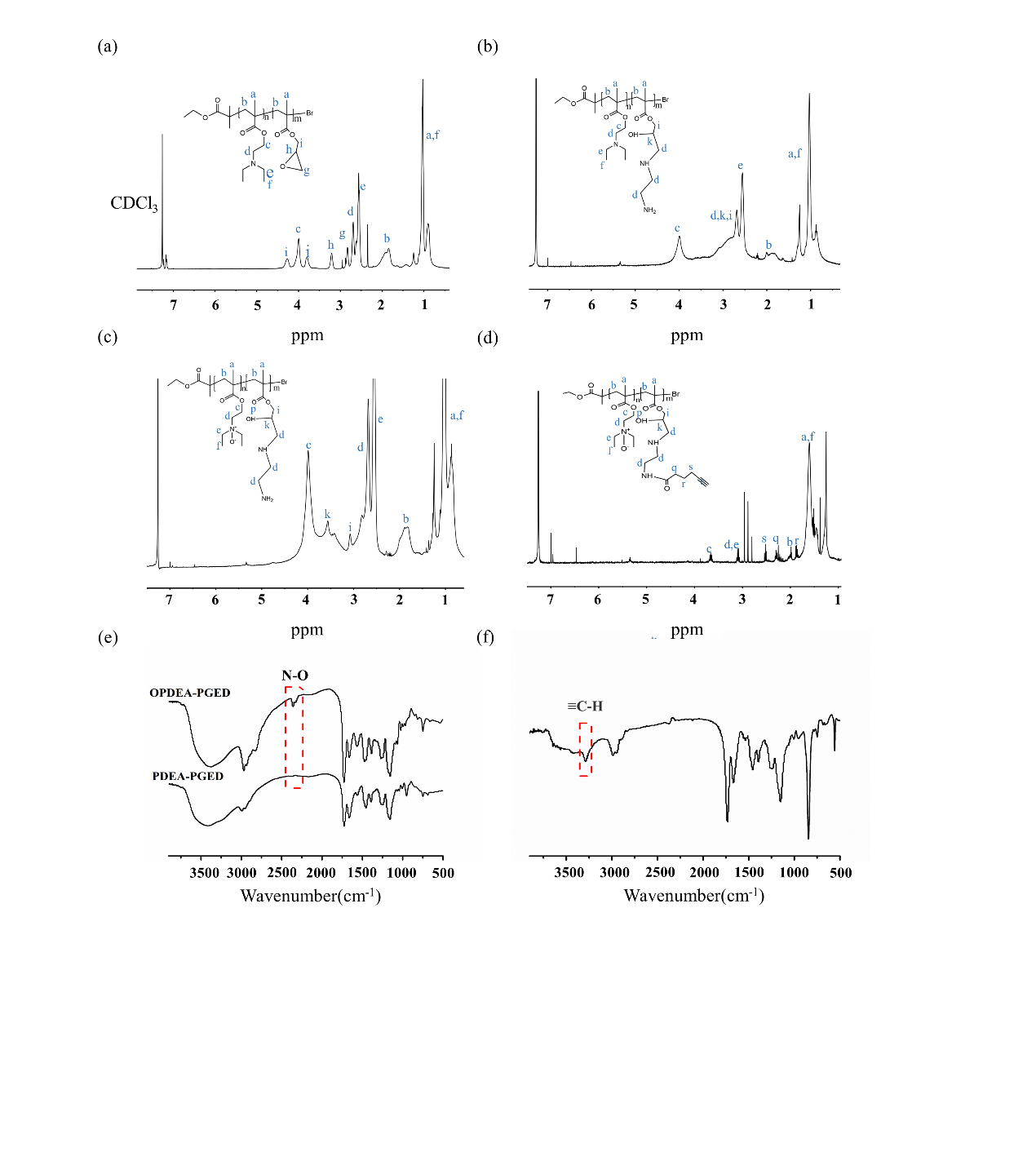


**Fig. S3** Characterization of polymers. **(a)** The ^1^H-NMR spectrum of PDEA-PGMA in CDCl_3_. **(b)** The ^1^H-NMR spectrum of PDEA-PGED in CDCl_3_. **(c)** The ^1^H-NMR spectrum of OPDEA-PGED in CDCl_3_. **(d)** The ^1^H-NMR spectrum of OPDEA-PGED-5HA in CDCl_3_. **(e)** The IR spectra of PDEA-PGED and OPDEA-PGED. (f) The IR spectrum of OPDEA-PGED-5HA.


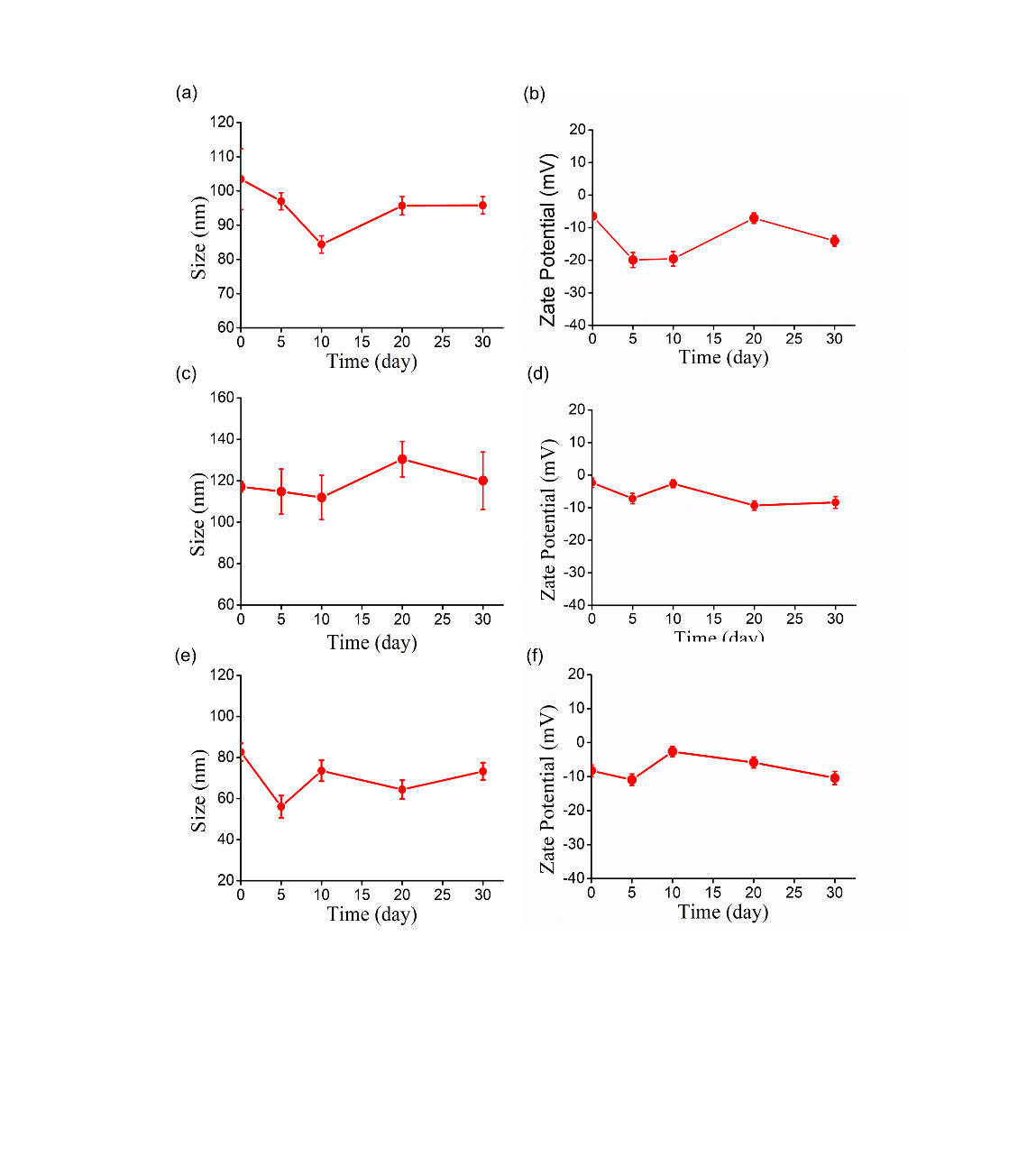


**Fig. S4** Stability of PPH, PPH@C and PPH@CR under saline and 4 °C within 30 days. **(a)** The particle size change of empty micelle PPH. **(b)** The zeta potential change of empty micelle PPH. **(c)** The particle size change of drug-loaded micelle PPH@C. **(d)** The zeta potential change of drug-loaded micelle PPH@C. **(e)** The particle size change of drug-loaded micelle PPH@CR. **(f)** The zeta potential change of drug-loaded micelle PPH@CR.

**Table S1.** The drug loading and encapsulation rate of PPH@C and PPH@CR

| **Sample** | **DL (%)** | **EE (%)** |
| --- | --- | --- |
| PPH@C | 14.47 ± 2.66 | 62.70 ± 11.55 |
| PPH@CR | 14.15 ± 2.33 (Cur) | 61.4 ± 5.11 (Cur) |
|  | 2.26 ± 1.42 (Rg3) | 12.29 ± 2.64 (Rg3) |

**Table S2**. The particle size changes of PPH, PPH@C and PPH@CR under saline and 4 °C within 30 days

| **Time (day)** | **PPH (nm)** | **PPH@C (nm)** | **PPH@CR (nm)** | |
| --- | --- | --- | --- | --- |
| 1 | 103.46 ± 8.88 | 117.15 ± 2.55 | | 82.66 ± 4.27 |
| 5 | 96.98 ± 2.49 | 114.81 ± 10.89 | | 56.06 ± 5.42 |
| 10 | 84.37 ± 2.54 | 111.96 ± 10.68 | | 73.56 ± 5.08 |
| 20 | 95.66 ± 2.66 | 130.42 ± 8.58 | | 64.41 ± 4.53 |
| 30 | 95.80 ± 2.53 | 120.06 ± 13.88 | | 73.24 ± 4.17 |

The data are presented as means ± SD (*n* = 3).

**Table S3.** The zeta potential changes of PPH, PPH@C and PPH@CR under saline and 4 °C within 30 days

| **Time (day)** | **PPH (mV)** | | **PPH@C (mV)** | **PPH@CR (mV)** |
| --- | --- | --- | --- | --- |
| 1 | | -6.45 ± 1.17 | -2.35 ± 1.45 | -8.24 ± 1.72 |
| 5 | | -19.92 ± 2.32 | -7.19 ± 1.59 | -10.93 ± 1.71 |
| 10 | | -19.55 ± 2.24 | -2.65 ± 1.18 | -2.65 ± 1.58 |
| 20 | | -7.10 ± 1.60 | -9.34 ± 1.42 | -5.85 ± 1.61 |
| 30 | | -14.06 ± 1.65 | -8.39 ± 1.80 | -10.41 ± 1.91 |

The data are presented as means ± standard deviation (*n* = 3).

**Table S4** The particle size and zeta potential changes of PPH@C and PPH@CR in different pH environments

| **Sample** | **Size (nm)** | **Zeta Potential (mV)** | |
| --- | --- | --- | --- |
| [PPH@C (pH=7.4)](mailto:PPH@C(pH=7.4))  PPH@C (pH=5.5) | 117.15 ± 2.55  105.31 ± 3.20 | | -2.35 ± 1.45  -25.34 ± 2.35 |
| PPH@CR (pH=7.4) | 82.66 ± 4.27 | | -8.04 ± 1.72 |
| PPH@CR (pH=5.5) | 55.21 ± 3.01 | -25.26 ± 3.20 | |

The data are presented as means ± standard deviation (n = 3).

### References:

1. Shen M, Li H, Yao S, Wu X, Liu S, Yang Q, et al. Shear stress and ROS-responsive biomimetic micelles for atherosclerosis via ROS consumption. Mater Sci Eng C Mater Biol Appl. 2021;126:112164.

2. Chen S, Zhong Y, Fan W, Xiang J, Wang G, Zhou Q, et al. Enhanced tumour penetration and prolonged circulation in blood of polyzwitterion-drug conjugates with cell-membrane affinity. Nat Biomed Eng. 2021;5:1019-37.

3. Luo K, Yang J, Kopeckova P, Kopecek J. Biodegradable Multiblock Poly[N-(2-hydroxypropyl)methacrylamide] via Reversible Addition-Fragmentation Chain Transfer Polymerization and Click Chemistry. Macromolecules. 2011;44:2481-8.

4. Liu Y, Chen, F, Zhang, K, Wang, Q, Chen, YW, Luo, XL. pH-Responsive reversibly cross-linked micelles by phenol–yne click via curcumin as a drug delivery system in cancer chemotherapy. Journal of Materials Chemistry B. 2019;7:3884-93.

5. Su R, Yan H, Jiang X, Zhang Y, Li P, Su W. Orange-red to NIR emissive carbon dots for antimicrobial, bioimaging and bacteria diagnosis. J Mater Chem B. 2022;10:1250-64.

6. Guo B, Yang F, Zhang L, Zhao Q, Wang W, Yin L, et al. Cuproptosis Induced by ROS Responsive Nanoparticles with Elesclomol and Copper Combined with alphaPD-L1 for Enhanced Cancer Immunotherapy. Adv Mater. 2023;35:e2212267.

7. Sun D, Zou Y, Song L, Han S, Yang H, Chu D, et al. A cyclodextrin-based nanoformulation achieves co-delivery of ginsenoside Rg3 and quercetin for chemo-immunotherapy in colorectal cancer. Acta Pharm Sin B. 2022;12:378-93.

8. Shen M, Yao S, Li S, Wu X, Liu S, Yang Q, et al. A ROS and shear stress dual-sensitive bionic system with cross-linked dendrimers for atherosclerosis therapy. Nanoscale. 2021;13:20013-27.

9. Xu Y, Zi Y, Lei J, Mo X, Shao Z, Wu Y, et al. pH-Responsive nanoparticles based on cholesterol/imidazole modified oxidized-starch for targeted anticancer drug delivery. Carbohydr Polym. 2020;233:115858.

10. Xu S, Feng Z, Zhang Y, Ni H, Liu Z, Wang D. pH-responsive Astragalus polysaccharide-loaded PLGA nanoparticles as an adjuvant system to improve immune responses. Int J Biol Macromol. 2022;222:1936-47.

11. Yin Y, Jiang H, Wang Y, Zhang L, Sun C, Xie P, et al. Self-Assembled Nanodelivery System with Rapamycin and Curcumin for Combined Photo-Chemotherapy of Breast Cancer. Pharmaceutics. 2023;15.

12. Han SJ, Sung N, Wang J, O'Malley BW, Lonard DM. Steroid receptor coactivator-3 inhibition generates breast cancer antitumor immune microenvironment. Breast Cancer Res. 2022;24:73.

13. Shen M, Jiang H, Zhao Y, Wu L, Yang H, Yao Y, et al. Shear Stress and ROS Dual-Responsive RBC-Hitchhiking Nanoparticles for Atherosclerosis Therapy. ACS Appl Mater Interfaces. 2023;15:43374-86.

14. Zakaria H, El Kurdi R, Patra D. Curcumin-PLGA based nanocapsule for the fluorescence spectroscopic detection of dopamine. RSC Adv. 2022;12:28245-53.

15. El Kurdi R, Patra D. Gold nanoparticles functionalized with Pluronic are viable optical probes for the determination of uric acid. Mikrochim Acta. 2018;185:185.

16. Ji P, Wang X, Yin J, Mou Y, Huang H, Ren Z. Selective delivery of curcumin to breast cancer cells by self-targeting apoferritin nanocages with pH-responsive and low toxicity. Drug Deliv. 2022;29:986-96.

17. Kumari M, Sharma N, Manchanda R, Gupta N, Syed A, Bahkali AH, Nimesh S. PGMD/curcumin nanoparticles for the treatment of breast cancer. Sci Rep. 2021;11:3824.

18. Yuan G, Zhang Y, Shao S, Zhou Z, Tang J, Xiang J, Shen Y. Tumor permeable self-delivery nanodrug targeting mitochondria for enhanced chemotherapy. J Control Release. 2023;361:792-802.

19. Feng X, Xu W, Liu J, Li D, Li G, Ding J, Chen X. Polypeptide nanoformulation-induced immunogenic cell death and remission of immunosuppression for enhanced chemoimmunotherapy. Sci Bull (Beijing). 2021;66:362-73.

20. Kroemer G, Galassi C, Zitvogel L, Galluzzi L. Immunogenic cell stress and death. Nat Immunol. 2022;23:487-500.

21. Li W, Yang J, Luo L, Jiang M, Qin B, Yin H, et al. Targeting photodynamic and photothermal therapy to the endoplasmic reticulum enhances immunogenic cancer cell death. Nat Commun. 2019;10:3349.

22. Wu H, Wei G, Luo L, Li L, Gao Y, Tan X, et al. Ginsenoside Rg3 nanoparticles with permeation enhancing based chitosan derivatives were encapsulated with doxorubicin by thermosensitive hydrogel and anti-cancer evaluation of peritumoral hydrogel injection combined with PD-L1 antibody. Biomater Res. 2022;26:77.
